# Alternative method to measure the VAT gap in the EU: Stochastic tax frontier model approach

**DOI:** 10.1371/journal.pone.0211317

**Published:** 2019-01-28

**Authors:** Danuse Nerudova, Marian Dobranschi

**Affiliations:** Mendel University of Brno, Faculty of Business and Economics, Department of Accounting and Taxation, Brno, Czech Republic; The Bucharest University of Economic Studies, ROMANIA

## Abstract

In this paper, we pursue an alternative method to measure the Value Added Tax gap in the European Union using the stochastic tax frontier model. We use the Value Added Tax total tax liability as the input to estimate the optimal frontier of the Value Added Tax, as well as to predict technical inefficiency. Using the latest innovations of the stochastic frontier approach, we aim to obtain the accurate size of the Value Added Tax gap in the EU-26 countries and contrast them with extant estimates. The obtained estimates of the Value Added Tax gap using the stochastic tax frontier model are different from the estimates produced by the top-down method to calculate the Value Added Tax gap in the EU. Moreover, the stochastic tax frontier approach allows us to disentangle the Value Added Tax gap, which is time dependent, from the persistent Value Added Tax gap, which is country specific. The stochastic tax frontier model allows us to test the effect of exogenous factors on the technical inefficiency of the Value Added Tax and propose appropriate policy recommendations.

## Introduction

Tax gap occurrence represents an ever-growing source of negative effects with multidimensional implications on public policy. Tax agencies and economists alike have raised concerns regarding the impact of the tax gap on the rule of law, public deficits, austerity measures and the supply of public goods and services. Tax gap measurement is worthwhile policy wise because it could provide essential information for a better understanding of tax compliance and its implications on tax policy. Moreover, tax gap estimation provides clues regarding a tax agency’s efficiency in collecting tax revenues and the needed resource allocation in order to address tax gap occurrence. The Value Added Tax (hereinafter VAT) represents one of the most important sources of tax revenue in the EU member states, currently constituting approximately one-fifth of total tax revenues. The member states face global challenges with tackling tax evasion and tax fraud in the area of direct as well as indirect taxation. Hence, the precise determination of VAT gap is a fundamental step in assessing the VAT efficiency and uncovering the true size of tax evasion and tax avoidance. The accuracy of the VAT gap represents a major issue for tax compliance analysis. The VAT gap is also critical for shaping policymakers’ strategy regarding the needed reforms to decrease tax evasion and tax avoidance.

The objective of this paper is to determine the VAT gap in EU countries using a nontraditional analytical method to both improve the accuracy of VAT gap estimates and provide relevant policy recommendations. To have more precise estimations, we propose a VAT gap calculation for the EU-26 member states that uses the stochastic tax frontier model (hereinafter STFM). The purpose of using this model is to provide an alternative method to calculate the VAT efficiency and the VAT gap and to compare the obtained output with the results of the top-down approach used by the reports of CASE [1, 2, 3, 4, 5].

The top-down approach determines the VAT gap by calculating the ratio between the VAT and the VAT total tax liability (hereinafter VTTL), whereas the STFM estimates the maximum level of tax revenues that a country can collect based on VTTL, assuming a certain level of technical inefficiency. Therefore, the estimates of the STFM are more relevant to policy than those of the top-down method. Beside the computational abilities of the STFM, the model generates impact estimations of other external factors on the VAT inefficiency term that are not included in the input set of variables. This alternative approach in the form of the STFM enables us to identify the measures necessary to increase the VAT efficiency and reduce the VAT gap in the EU. The stochastic tax frontier model allows us to estimate the effect of exogenous factors on the technical inefficiency of the Value Added Tax and propose appropriate policy recommendations, i.e., its application enables the formulation of more targeted regulations to increase VAT efficiency collection.

Literature that has addressed the issue of tax gap and especially the VAT gap is scarce. Moreover, to our knowledge, Brun and Diakite [6] is the only research paper that analyses VAT efficiency using the stochastic frontier model. The authors analyze the overall tax capacity and tax effort as well as VAT capacity and VAT effort in non-resource developing countries. The main criticism of this paper arises from the choice of inputs employed to determine the VAT capacity and the VAT effort. The study uses GDP per capita, agriculture share in GDP, the degree of the economy’s openness, and the resource revenues as a percentage of GDP. While these independent variables might bear some explanatory power on estimating a country’s general tax capacity and tax effort, we assume that specific inputs of VAT revenues–namely, the VAT total tax liability calculated by CASE [1, 2, 3, 4, 5]–should be used to analyze VAT output efficiency. Our paper contributes to the current literature by adopting stochastic frontier approaches to calculate the VAT gap and to identify the main external factors that affect the VAT efficiency. The use of the STFM allows us to determine the sign of the impact and the magnitude of the exogenous determinants on the VAT gap in the EU.

The methodology used to analyze the VAT efficiency and determine the VAT gap adopts two different stochastic frontier models: the time-varying inefficiency model of Battese and Coelli [7] and the model that separates the country effects from persistent inefficiency and time-varying inefficiency proposed by Kumbhakar, Lien and Hardaker [8].

The paper is organized as follows. In the first section, a review of the literature regarding the concept of tax gap is presented; in the second section, we present the current situation regarding the VAT gap calculation; in the third section, the theoretical background of stochastic frontier models is reviewed; in the fourth section, the data and methodology are described; in the fifth section, the results of the adopted stochastic tax frontier models are presented; in the sixth section, the limitation of the paper are discussed; the final section comprises the concluding remarks.

## Tax gap–literature review

The complexity of tax compliance and research about new methods to reduce tax noncompliance have preoccupied tax practitioners and economists alike. Andreoni, Erard and Feinstein [9] consider tax compliance an issue of public finance, tax law enforcement, the tax authority’s organizational design, ethics and tax morale or a complex combination of all of them. The authors perform an extensive review of the theoretical and empirical literature regarding tax compliance. Andreoni, Erard and Feinstein [9] define tax gap as a popular indicator of the size of tax evasion; it represents the difference between the taxes collected and taxes owed to the public budget. The authors indicate that theoretical models devised to explain the complex issue of tax compliance have not been fully used by the empirical literature. Andreoni, Erard and Feinstein [9] underline the need to explore the influences of psychological, moral and social factors on tax compliance. The inclusion of these factors could greatly improve the economic models related to tax noncompliance and tax gap estimation. Moreover, Andreoni, Erard and Feinstein [9] stress that the analysis of institutional framework complexity could become another method to measure tax compliance and tax gap.

From the behavioral point of view, Elffers, Weigl and Hessing [10] define tax evasion behavior as a form of tax cheating. It represents a premeditated act of noncompliance which leads to less tax paid than is actually owed to the government. Kirchler [11] considers tax evasion a form of deliberate underreporting of income or claiming unwarranted tax deductions. Tax evasion does not include unintended noncompliance, which could result from memory lapses, errors in calculation or inadequate knowledge of tax law. Kirchler [11] underlines that tax noncompliance is a broad concept that refers to intentional and unintentional failures to meet tax obligations. The author stresses that tax avoidance represents a legal form of reducing tax liability by exploiting loopholes in tax law and the “creative designing” of the taxpayer’s own income and deductions. On the opposite side, tax evasion pertains to illegal practices to reduce the amount of taxes paid. Kirchler [11] stresses that tax noncompliance in its various forms (e.g., tax avoidance and tax evasion) should be approached because it lowers tax revenues collected for public budgets, affects the provision of public goods and services, undermines tax effects on fair income redistribution, deteriorates the feeling of fair treatment and, in general, enhances disrespect of the law.

Warren and McManus [12] define tax gap as the amount of tax revenues legally owed to the government that are not collected. The authors divide tax gap into three components: underreporting income, underpayment of income and non-filing of tax returns. The most important factors of tax gap arise from uncollected taxes due to unintentional errors, the underground economy and illegal activities. Other factors that contribute to tax gap formation are due to the dissatisfaction of taxpayers with their government, public spending policy, corruption and even a high level of tax law complexity, which may lead to poor tax compliance. According to Warren and McManus [12], the measurement of tax gap could provide essential information for tax agencies in order to estimate the level of tax compliance. Warren and McManus [12] stress that there are three types of benefits which arise from tax gap measurement: first, it allows the identification of sources and levels of noncompliance; second, tax gap analysis improves the efficiency of resource allocation within tax revenue agencies and third, tax gap estimation serves as a measure of tax authority effectiveness in collecting tax revenues.

The institutional definition of tax gap has been given by the IRS [13] and HMRC [14], and it represents the difference between what the taxpayers should pay and what they actually pay on timely basis. HMRC [14] underlines that measuring the tax gap provides useful clues to tax agents for understanding the size and nature of tax noncompliance. Estimating the tax gap, regardless of which type of taxation is focused on (e.g., direct or indirect taxes), provides information about how tax noncompliance occurs and how can tax agencies address the issue. Additionally, determining the level of unpaid taxes presents an image of overall efficiency of the tax authority. HMRC [14] points out that there are several factors that lead to tax gap occurrence, such as: simple human error in calculating the owed taxes, errors in submitted tax returns, errors in the legal interpretation of tax laws, tax evasion, tax avoidance and criminal attacks on the tax system. Moreover, the tax gap can appear in cases of business insolvency where the outstanding tax revenues cannot be collected.

Murphy [15] stresses that the size of the tax gap has a direct impact on public spending policy. A high tax gap increases the public deficit and decreases the supply of public goods and services, which affects the quality of life and the well-being of those individuals who depend on governmental services. Murphy [15] divides the tax gap into three parts: tax debt or those revenues which delay tax payment; tax avoidance or the lawful practices of a taxpayer used to minimize their tax burden and tax evasion, represented by the illegal and deliberate actions of taxpayers which fail to declare their taxable income and claim undeserved tax deductions. According to Murphy [15], tax evasion can be further classified into: the shadow economy or those economic activities that are not recorded and hidden in order to avoid taxation; taxes lost due to fraudulent or criminal activities; offshore tax evasion and tax evasion from investment and rental income. The author suggests several appropriate solutions to tackle tax noncompliance in the UK, such as: implementation of proper anti-avoidance rules in the tax law; introduction of country-by-country reporting of multinational enterprises in order to reduce corporate tax avoidance; reform the taxation of small companies to enforce regulation regarding corporate taxation and increase the staffing of UK tax agencies to improve their efficiency.

The empirical literature concerned with tax gap measurement can be divided into two distinct groups. The first group of studies is represented by the control and audit methods based on a large sample of representative taxpayers where the results obtained are generalized to a whole population. Gemmell and Hasseldine [16] label this first group of methods as micro-methods. According to United States Government Accountability Office (GAO [17]) this type of method is especially used by the US Internal Revenue Service to measure tax noncompliance and calculate tax gap. The representative audit method used by the IRS is the Taxpayer Compliance Measurement Program (TCMP), used to estimate tax gap in the US.

The second group of empirical studies relies on macro-methods. According to Raczkowski [18], the macro-methods are considered indirect approaches to measuring the tax gap. Gemmell and Hasseldine [16] label the second group of empirical studies as discrepancy methods. The discrepancy methods are based on the difference between a reported income and an independent measure of income, which assumes the inclusion of a hidden economy. Gemmell and Hasseldine [16] point out that the main weakness of discrepancy methods based on macroeconomic variables is the high level of aggregation. Consequently, these methods are unable to provide accurate levels of tax gap for different types of taxes. The main approaches to indirectly estimate tax gap resort to the national income-expenditure method, single indicator methods and multiple indicator methods (Multiple Indicator Multiple Causes model–MIMIC).

Giles [19] adopts the MIMIC model to predict the size of the underground economy in the New Zealand. The structural MIMIC model treats the hidden economy as a “latent variable” and uses a currency demand model to determine the size of underground economy. Once the size of the underground economy is estimated, the author calculates the size of tax gap in New Zealand. Giles [19] finds that the size of the hidden economy in New Zealand is between 6.8% and 11.3% of measured GDP on average. Consequently, the author estimates an overall tax gap that varies between 6.4% and 10.2% of GDP for 1968–1994 period. Giles [19] concludes that tax gap as a percentage of total tax liability is a monotonic increasing function of hidden economy to GDP ratio because underground activities remain untaxed and create a gap between actual and potential tax revenues.

Another study that uses the MIMIC model was done by Raczkowski [18]. The author uses this model to calculate the overall tax gap in the EU member states. Raczkowski [18] concludes that the average overall tax gap in the EU was 10.7% of GDP in 2014. The highest tax gaps were found in Italy, with 13.8% of GDP, Estonia, with tax gap of 13.6% of GDP, and Romania, with a tax gap of 12.9% of GDP. On the opposite side, the lowest tax gaps were found in Luxembourg, with 1.7% of GDP, and Ireland, with an overall tax gap of 3.2% of GDP.

The literature has raised concerns regarding the reliability of conventional methods to measure tax gaps. Gemmell and Hasseldine [16] underline that both micro- and macro-methods used to calculate tax gap fail to incorporate the behavioral responses of taxpayers in their estimation methodology, leading to less reliable tax gap estimates. It is important to note that there is an extensive literature concerned with taxpayer behavioral economic modeling. Allingham and Sandmo [20] were the first economists to devise a theoretical behavioral economic model to analyze tax compliance. The behavioral model studies the interaction between the tax authority, which sets the auditing rules, and the taxpayers, who engage in tax evasion in response. Reckers, Sanders and Roark [21] analyze the behavioral factors that affect tax compliance. The reason behind this study was to prove that high tax rates are not the only factor playing a major role in tax compliance, but ethical beliefs also have a strong influence on tax compliance. Reckers, Sanders and Roark [21] found that tax ethics are highly significant when a taxpayer decides to evade taxation and represents the “missing variable” of tax decision-making models. O’Doherty [22] uses the model by Allingham and Sandmo [20] to determine tax administration best-response audit strategy in a context with heterogeneous taxpayers and imperfect information. O’Doherty [22] concludes that tax administrations’ best response to a range of behavior of tax evaders is an adaptive learning approach. This conclusion is in line with the “predictive analytics” practice used by the OECD tax authorities.

In terms of behavioral economic models, Feldstein [23, 24] analyzed taxpayer behavioral responses to changes in tax rates. Feldstein [24] terms this responsiveness as the elasticity of taxable income. Based on Feldstein’s [24] seminal contributions, Gemmell and Hasseldine [25] underline that failure to include the behavioral responses of taxpayers into the conventional methods used to calculate the tax gap tend to overestimate tax compliance.

Malik, Mihm and Timme [26] conduct a laboratory experiment in order to analyze the impact of Anti-Avoidance Tax Rules (hereinafter AAR) on overall tax compliance. The scope of AAR is to reduce aggressive tax avoidance practices and increase the collection of tax revenues. This set of principle-based tax rules allows tax authorities to differentiate between responsible tax planning and aggressive tax avoidance. Malik, Mihm and Timme [26] consider that the size of tax gap depends strongly on the interaction between citizens as taxpayers and the government, the perception of the tax law and the perception of past fiscal policies. Malik, Mihm and Timme [26] found that the adoption of AARs to increase tax compliance leads to a substitution effect between tax avoidance and tax evasion. While AARs do achieve some reduction in tax avoidance, Malik, Mihm and Timme [26] observe that tax evasion tends to increase in this context. The authors conclude that the substitution effect does decrease the effectiveness of AARs in lowering the tax gap. Even if AARs decrease aggressive tax planning practices, the potential increase in tax revenues is offset by the increase in tax evasion as a response to AAR’s introduction.

The literature points out that there is an increasing interest from various countries in measuring tax gap. Barthelemy [27] notes that VAT fraud is regularly investigated in France. Sweden is also preoccupied with estimating tax gap, including tax noncompliance in the case of VAT. The tax gap has also been analyzed in the UK and the US. The UK has measured the size of VAT gap on an annual basis starting in 2001.

In terms of tax gap size, the IRS [28] estimates that the overall tax gap in the US for tax years 2008–2010 was 458 billion USD annually. The IRS report [28] divides tax gap into three main categories: non-filing tax gap or those taxes not paid due to not filing tax reports at all or filling after the due date; underreporting tax gap or those taxes not paid by misreporting the true tax liability and underpayment tax gap or taxes reported and not paid in a timely manner. The IRS [28] concludes that the overall net tax gap was 16.3% of total tax liability in the US in analyzed tax years. HRMC [14] estimated that the overall tax gap in the UK for 2016–2017 amounted to 33 billion GBP. This means that the overall tax gap was 5.7% of total tax liability. In the case of VAT, HMRC [14] estimated that VAT gap amounted to between 8.9% to 12.5% of total VAT liability for the 2016–2017 tax years. Murphy [15] estimated that the tax gap in the UK for the 2014–2015 tax years was at least 122 billion GBP. A large share of the tax gap in the UK is attributed to tax evasion. Murphy [15] points out that if the tax revenues representing the estimated tax gap could be recovered, this could easily cover 90% of total public spending on the National Health System (hereinafter NHS) in the UK.

## The current developments in VAT gap calculation in the EU

The European Commission [29] underscored that the VAT gap is the only tax gap estimated for the all EU member countries; there are only 11 EU member states that calculate tax gaps for other taxes: the Czech Republic, Estonia, Finland, Germany, Italy, Latvia, Poland, Portugal, Slovakia, Slovenia and the United Kingdom. According to OECD [30], concern regarding the determination of the tax gap is greatly overlooked; only 43% of the analyzed tax agencies have tax gap measures. Moreover, Murphy [31] stresses that in addition to the fact that the EU states tend to disregard tax gap measurements, there are also great differences in the methodologies adopted by the EU states that do engage in tax gap determination. Existent literature, such as Ebrill et al. [32] and OECD [33], propose a more sophisticated measure of VAT gap to calculate the VAT gap named c-efficiency or the VAT Revenue Ratio (hereinafter VRR). This method of determining the VAT gap is defined as the ratio of actual VAT revenues to the VAT revenues that would be collected if the VAT is applied at the standard rate on the entire potential tax base in a pure VAT regime (OECD, [33]). The potential VAT base includes all supplies of goods and services and intangibles consumed by businesses, individuals or governments and other entities that act as businesses like non-profit organizations (hereinafter NGOs). This method assumes a pure VAT regime where only a unique VAT rate is applied, and all reduced VAT rates are excluded. The aim of VRR is to provide a comparative measure of a country’s ability to effectively secure the potential tax base. The VRR measures the difference between the actual VAT revenues and the theoretical VAT revenues that would have been obtained had the VAT been applied at a standard rate for the entire tax base.

OECD [33] uses several components to build the potential VAT base: private final consumption expenditures of households; final consumption expenditures of NGOs; and final consumption expenditures of general government. One important adjustment recommended by OECD [33] is related to the construction of the potential VAT base for VRR calculation: the exclusion of VAT from the market prices. The national account data measure the consumption at market prices including the VAT. Thus, the VAT revenues should be removed from this amount since the theoretical tax base for VAT should not include the tax itself. According to CASE [1], the weakness of the VRR method stems from the strong assumption of moving the benchmark (or imposing the standard VAT rate on all consumption) and would not affect the level of composition or the level of consumption. Moreover, CASE [1] stresses that the VRR model does not discriminate between the VAT gap that is due to policy efficiency or compliance efficiency. Therefore, the VRR can be decomposed into the policy gap–or the VAT gap that is due to the reduced VAT rate applied–and VAT exemptions that reduce the VAT revenues collected. The second part of VRR can result from the compliance gap or the difference between actual VAT revenues and the potential VAT revenues that could have been collected had no taxpayer been involved in any tax evasion or tax avoidance.

In terms of individual country studies, the tax agencies of Sweden and the United Kingdom have been preoccupied with VAT gap estimation. The Swedish Tax Agency (hereinafter STA) analyzed the tax gap changes in Sweden between 2007 and 2012. STA considers tax gap to be the difference between the tax that would have been determined if all taxpayers had reported all their gainful activities and transactions and the tax determined in practice following the STA’s control procedures. According to Skatteverket [34], the STA has a systematic approach to measuring the level of tax noncompliance and the size of the tax gap in Sweden. The first method adopted by STA is building a Tax Information Map. The scope of such an approach is to identify and isolate those factors that affect tax compliance. Moreover, the Tax Information Map shows to what extent the STA has access to tax information (i.e., information needed to assess the tax base and calculate tax revenues) “over and above” the information (i.e., tax returns) provided by the taxpayers. The Tax Information Map has eight levels of information, where the eighth level represents the highest level of tax information that STA has access to; and it is considered the least susceptible to errors in calculating the tax owed. This method determines the risk of tax gap occurrence and its variation between different categories of taxes in Sweden. According to Skatteverket [34], VAT occupies the fourth position on the Tax Information Map, meaning that there is not sufficient tax information to determine an exact tax base.

The second method used by STA, as underlined by Skatteverket [34], is the top-down method. This method is used especially for VAT discrepancy calculation or the measurement of VAT gap. The top-down method adopted by STA calculates the real size or the theoretically correct VAT based on data obtained from national accounts. To calculate the theoretical VAT, STA focuses on household final consumption expenditure, intermediate consumption and fixed gross investment, which is not subject to VAT deductions or VAT reimbursement. The VAT gap is obtained from the difference between theoretical VAT and the VAT actually collected for the public budget. Another method used by STA to calculate VAT gap is the measurement of hidden incomes in the household sector. This method calculates the difference between incomes used and the actual incomes reported.

Skatteverket [34] concluded that the VAT gap in Sweden between 1993 and 2002 was only 1.3% of the theoretically correct VAT. Reckon [35] found that between 2000–2006, Sweden had a VAT gap averaging 3%. CASE [1] adopted the same top-down method to calculate VAT gap in the EU and obtained a higher level of VAT gap in Sweden, which amounted to 5%, in comparison with Reckon [35]. Skatteverket [34] attributes this difference to different sets of data used by CASE [1]. The calculations made by CASE [1] have been adapted to country-specific conditions and have used more disaggregated data regarding the theoretical size of VAT. The conclusion of CASE [1] and Skatteverket [34] regarding the VAT gap in Sweden is that it follows a steady downward trend, and it is the lowest gap when compared with other EU countries.

The United Kingdom’s tax agency, namely, HM Revenue & Customs (hereinafter HRMC), has been preoccupied with the overall tax gap and different categories of tax gap measurement since 2005. The VAT gap is also calculated by HMRC on a yearly basis in the UK. The latest estimates of overall tax gap and VAT gap are done by HMRC [14]. The British tax agency uses distinct methods to calculate tax gaps, such as: data matching by comparing related tax revenues databases; top-down methods and bottom-up methods that include random enquiries, management information and experimental procedures to estimate tax gap levels where data is limited.

HMRC [14] uses the top-down method to calculate the VAT gap. The first step is calculating the gross VAT total theoretical liability based on the total expenditure of the British economy that is liable to VAT. The data regarding the VAT base is taken from Office for National Statistics (hereinafter ONS). The second step is the gross VTTL adjustments by tax and other deductions in order to determine the net VTTL. Once the net VTTL is calculated, it is compared with the actual VAT revenues collected. The difference between theoretical and actual VAT represents the VAT gap. It is important to mention that each HMRC report provides the revised estimations of tax gap for past tax years. This revision or recalculation of historical tax gaps from 2005 onward is due to the updated data and updated projections and methodological improvements adopted by HMRC.

The main limitation of HMRC [14] estimates of VAT gap and their accuracy is related to the method adopted. Because the VTTL calculation is based on survey and other data analysis, some of these data also includes assumptions and adjustments that produce random and systematic variations of VAT gap estimates. Moreover, HMRC [14] calculates the VTTL based on small elements of forecasting regarding spending data, which produces variations in the VTTL estimation.

HMRC [14] underlines that it is not possible to produce reliable confidence intervals for VAT gap estimates. The main reason for this setback is because the VTTL is based on ONS data derived from sample surveys that also incorporate sampling and non-sampling errors. Observing the VAT gap estimates produced by HMRC [14], there is a clear downward trend in VAT revenue losses between 2005–2015. On average, HMRC [14] estimates that the VAT gap in the UK was 8.9% for the period of 2005–2015, and the UK registered a VAT gap of 9.95% in 2015.

In addition to the country level estimation of the VAT gap, the European Commission has been involved in funding research institutes that would calculate the VAT gap for the entire EU.

Reckon [35] performed a study commissioned by the European Commission to measure and analyze the VAT gap for the EU member states between 2000–2006. The authors adopted the top-down method in estimating the VAT gap. The top-down method uses the data for each EU member state’s national accounts to calculate theoretical VAT liability and compare it to the actual VAT revenues collected. The limitations of the study arise from the high level of aggregation. The VAT gap estimate by Reckon [35] is for a whole economy and cannot be disaggregated by sector in order to identify which sectors contribute to VAT gap occurrence and which activities are more prone to VAT fraud. Reckon [35] found that for the EU-24, except for Cyprus due to lack of data, the average VAT gap in 2006 was 12% of theoretical VAT liability. The worst performing EU country was Greece with a 30% VAT gap, and the most efficient EU country in collecting VAT revenues was Luxembourg with a VAT gap of only 1%. Comparing these data with CASE reports [1, 4, 5] and Skatteverket [34], Sweden is therefore not the most efficient country in collecting VAT revenues. Reckon [35] explains the differences in VAT gap levels between their results and annual VAT gap estimates done by each EU country’s tax agency due to the national tax administration’s access to more detailed data sources. Beside the VAT gap estimation for EU-24, Reckon [35] also performed an econometric analysis, which was intended to explain the nature and causes of VAT gap in the EU-24. The VAT gap estimates were regressed to a set of macroeconomic, social, fiscal, cultural and institutional explanatory variables. The authors found that one of the most significant factors in enhancing the VAT gap occurrence in the EU-24 was the perceived level of corruption. As the perceived level of corruption decreases, Reckon [35] found that VAT gap also tends to decrease.

The overall conclusion reached by Reckon [35] shows that there is a downward trend in VAT gap, with substantial decreases in Luxembourg, Poland and Slovenia between 2000–2006. Belgium, Denmark, Spain, Ireland, the Netherlands and Sweden showed a less pronounced decrease in VAT gap in the analyzed period. Reckon [35] found that in Austria, Germany, France, Finland and the UK there was a relatively stable VAT gap with small yearly fluctuations. On the other hand, Greece, Hungary and Lithuania showed an increasing trend in VAT gap between 2000–2006.

The current source regarding the size of the VAT gap in the EU is contained in the reports of CASE [1, 2, 3, 4, 5], commissioned by the Directorate-General for Taxation and Customs Union. Since there are no alternative studies that determine the size of the VAT gap, the CASE estimates represent the common landmark used to assess tax evasion and tax avoidance in the EU. The reports produced by CASE [1, 2, 3, 4, 5] are the only studies since the works of Reckon [35] that estimate the VAT gap on a yearly basis for all the EU countries. The aim of the CASE studies is to update and refine the VAT gap estimates of Reckon [35] and include the new EU member countries such as Cyprus, Bulgaria and Romania. CASE [1] follows the same methodology initially proposed by Reckon [35], using a top-down method to calculate the total theoretical liability for VAT and compare it to the actual VAT revenues in order to determine the level of VAT gap. CASE [1] does not include Croatia, since it became full EU member in 2013 and Cyprus is excluded due to the lack of data regarding national accounts.

According to CASE [1, 2, 3, 4, 5], the VAT gap is the difference between theoretical tax liability and the actual revenues collected. The VAT theoretical tax liability (VTTL) is calculated using a top-down approach according to tax law. The top-down method for calculating the VAT gap applies the VAT rates from each of the 26 EU countries to six main determinants of VAT revenues: final consumption of households; final consumption of governments; final consumption of nonprofit institutions serving households (hereinafter NPISH); intermediate consumption; gross fixed capital formation (hereinafter GFCF); and country-specific adjustments such as rebates, reductions, and VAT exemptions. The main criticism of this approach is that a top-down method based on national account data requires some degree of approximation to calculate the VTTL. CASE [1] computes the VTTL as the sum of two major components: the VAT paid by final consumers and the VAT paid by producers. The final consumers pay VAT for taxable goods and services, and producers pay VAT for inputs used to produce goods and services that are nontaxable or are exempt from taxation. The data used to build the VTTL by CASE [1] is provided by the World Input-Output Database (hereinafter WIOD).

The most notable contribution to the literature by the CASE [1, 2, 3, 4, 5] reports is the careful and systematic procedures adopted to calculate the total theoretical liability of VAT for each EU member state. The authors employed a disaggregated top-down method by imposing appropriate VAT rates (i.e., standard and reduced VAT rates when required) to a segmented final consumption tax base. Moreover, CASE [1] took into account the required VAT reductions, exemptions and VAT rebates granted by each EU state that would reduce the VTTL and thus VAT revenues. The complex task of matching relevant tax bases with VAT rates is based on the most detailed possible consumption database using national accounts, supply-use tables and household survey data.

Another contribution of CASE [1, 2, 3, 4, 5] to the literature is the decomposition of VAT gap into the compliance and the policy gap. Keen [36] defines the policy gap as a normalized measure of tax expenditures under VAT compared to a relative single VAT rate and final consumption under full tax compliance. Since only a few EU countries impose a single VAT rate on all consumption, the policy gap in VAT gap plays an important role. The impact of rate gap can be measured by taking into account the differences between standard and reduced VAT rates. The exemption gap can be deducted as the difference between rate gap and policy gap.

The compliance gap is defined by CASE [1] as the difference (i.e., the VAT gap) between the actual VAT revenues and the theoretical VAT revenues that would be collected if there was perfect compliance with the tax law. According to CASE [1], the VAT gap resulting from the policy gap represents the uncollected VAT revenues due to the EU member states’ different VAT rates on several groups of goods and services (e.g., foodstuffs, pharmaceutic products, baby food, etc.). Therefore, the policy gap is the difference between the actual VAT revenues and the theoretical VAT revenues that would be collected if all the EU countries imposed a standard VAT rate on all goods and services. CASE [1, 2, 3, 4, 5] further decomposes the policy gap into the rate gap and the exemption gap. The VAT rate gap is assumed to occur due to losses of VAT revenues when member states impose reduced rates on selected goods, and the VAT exemption gap represents those VAT revenues lost to a VAT exemption granted by EU countries. CASE [1] underlines that policy and compliance gaps for VAT are not independent elements. The VAT gap produced by policy gaps, such as reduced VAT rates, exemptions or thresholds of the VAT, make tax compliance more difficult and increase efforts to determine the exact VAT liability. CASE [1] calculates the compliance VAT gap or the VAT gap measured by the difference between the VTTL and the actual VAT collected.

CASE [1] underlines that policy and compliance gaps for VAT are not independent elements. The VAT gap produced by policy gaps, such as a reduced VAT rates regime, exemptions or thresholds of the VAT, make tax compliance more difficult and increase the efforts to determine the exact VAT liability. CASE [1] calculates the compliance VAT gap or VAT gap measured by the difference between the VTTL and the actual VAT collected.

Another useful approach used by CASE [1, 5] is the use of an econometric analysis to investigate the factors that enhance the VAT gap in the EU. CASE [1] regressed the VAT gap estimates obtained from the top-down method to output gap, unemployment rate, standard VAT rate, imports expressed as percent of GDP, corruption perception level and EU accession. The EU accession explanatory variable was included in order to control the effects of a state accession on tax enforcement and changes into the tax design. CASE [1] found that unemployment does increase the VAT gap in the EU, and EU accession tends to decrease the level of tax noncompliance. In comparison with Reckon’s [35] results, CASE [1] found that the corruption perception level is not statistically significant in explaining the VAT gap evolution.

CASE [5] also performed an econometric analysis of VAT gap determinants. However, the econometric approach is substantially different from CASE [1]. The latest report from CASE combine macroeconomic explanatory variables with proxies that attempt to capture the behavioral factors that enhance the VAT gap in the EU. CASE [5] uses behavioral proxies related to buyers, sellers and the tax administration. The results obtained by CASE [5] show that unemployment has a positive impact on the VAT gap. The scale of tax administration expressed in administrative expenditure as a percent of GDP is efficient in reducing the VAT gap if it does not exceed the level of 5.5% of GDP.

CASE [4] found that the average VAT gap at the EU level is 12.77%, with the highest levels of the VAT gap in Romania (37.18%), Slovakia (29.39%), Greece (28.27%) and Lithuania (26.41%) and the lowest levels of VAT gap in Sweden (-1.42%), Spain (3.52%) and Slovenia (5.52%).

It is important to underline that CASE [5] also published the revised estimates of VAT gap in the EU countries for 2015, which are significantly different from the estimates produced by CASE [4]. The previous report (e.g., CASE [4]) estimated that Sweden had a negative VAT gap (i.e., -1.42%) in 2015 and that it was the most efficient country at collecting VAT revenues in the EU, while CASE [5] revised the VAT gap estimates for 2015 and found that Sweden had a VAT gap of 3.51%. Moreover, CASE [5] found that Luxembourg was the most efficient country at collecting VAT revenues in 2015, with a VAT gap of 2.28% in comparison to CASE [4] which estimated Luxembourg’s VAT gap for 2015 as 5.56%. According to CASE [5], Luxembourg had the lowest VAT gap in 2016 at only 0.85% of VTTL, followed by Croatia at 1.5% and Sweden with 1.08%. The worst performing EU countries in terms of VAT gap in 2016 were Romania with 35.88%, Greece with 29.22%, Italy with 25.90% and Slovakia with 25.68% of VTTL. The overall conclusion of CASE [5] is that there was a general decline in the VAT gap in the EU; the average VAT gap in the EU-28 was 147 billion EUR in 2016 compared with 157 billion EUR in 2015.

The main limitation of the top-down method used to estimate the VAT gap stems from the relative accuracy of data used to construct the VAT tax base and the total theoretical VAT liability. The large differences in estimates obtained by CASE [4, 5] and HMRC [14] underline the weak reliability of the top-down method to accurately calculate the VAT gap in the EU. Moreover, the econometric analysis attempted by Reckon [35] and CASE [1, 5] can also be questioned. If the dependent variables, namely, the VAT gap levels, are not accurate, then the estimation of VAT gap determinates can lead to biased estimates. The stochastic tax frontier model represents an appropriate alternative to the top-down method for estimating VAT gap. Additionally, because the STFM is more computationally powerful, it can estimate the level of VAT inefficiency and the impact of the main determinant factors on VAT gap in one step.

## The stochastic tax frontier model–theoretical background

Aigner, Lovell and Schmidt [37] are the creators of the stochastic frontier model (hereinafter SFM). The difference between regular regression estimation and SFM is the decomposition of the error term into two distinct terms, *v*_*i*_ and *u*_*i*_. *v*_*i*_ represents the white noise or those factors influencing tax revenue collection that are outside of the country’s control, and *u*_*i*_ represents the inefficiency or the “failure” to achieve the maximum amount of output. The inefficiency *u*_*i*_ is a nonpositive term that determines the frontier production function.

Alfirman [38] was the first economist to introduce the concept of the stochastic tax frontier model as a production frontier used to analyze a country’s tax efficiency. A tax frontier function estimates the maximum amount of tax revenues that a country can collect from a given set of inputs. Alfirman [38] analyzes the tax potential in Indonesia using the following inputs to estimate the stochastic tax frontier function: the level of income, labor force participation, high school students per capita, the share of agriculture in overall GDP and the degree of openness of the economy. Alfirman [38] stresses that one of the limitations of this model is the questionable set of inputs chosen to estimate the tax frontier. While a production frontier from the classic stochastic frontier model is clearly determined by traditional inputs such as labor, capital and materials, there is less agreement on which of the economic, social, demographic and institutional inputs are more appropriate to estimate a country’s tax potential.

Pessino and Fenochietto [39] and Fenochietto and Pessino [40] have also adopted a model of stochastic tax frontier to analyze the tax effort and tax capacity in 96 world countries. The tax effort is defined as the gap between the maximum tax revenues that a country can collect based on its economic, social, institutional and demographic conditions and the current level of tax revenues collected. The tax potential is the maximum level of tax revenues that a country can collect in the absence of inefficiencies such as tax evasion and tax avoidance. The estimation strategy used by Pessino and Fenochietto [39] follows the model proposed by Battese and Coelli [7] and is applied to panel data to capture the effect of time-varying variables on tax effort and tax potential. Pessino and Fenochietto [39] use the following variables as inputs in estimating tax effort and tax capacity by the SFM method: GDP per capita as an indicator of development, openness of the economy (% of GDP), income distribution indicator (i.e., GINI), share of the agricultural sector in total GDP and the corruption level.

Cyan, Martinez-Vasquez and Vulovic [41] review two different methods used in the literature to measure tax effort: the traditional regression approach and the stochastic production frontier model that allows researchers to identify potential determinants of tax collection inefficiency. The authors note that the STFM is superior to the traditional regression approach because it is able to identify the weak links in tax administration that reduce tax collection efficiency. Cyan, Martinez-Vasquez and Vulovic [41] propose a third method to measure the tax effort by determining tax gap using the deviations between a country’s desired tax revenues as shown by its current choice of public expenditure and the country’s actual tax revenues accrued to the public budget. Therefore, the public deficit could be used as a measure of the tax gap between the preferred level of public expenditure and the current level of taxation. The evaluation of this method and the estimation of tax effort using this particular relationship offers the advantage of assessing the preference of a country regarding the size of public sector. Cyan, Martinez-Vasquez and Vulovic [41] calculate the tax effort of 94 countries using panel data analysis. The results obtained by the authors show that the tax effort obtained from the SFM method is 10% smaller than the tax effort obtained using traditional fixed effect regression analysis. The authors’ explanation for the 10% difference focuses on the benchmark chosen by each method. While the SFM method chooses the best performance among the analyzed countries, the regression analysis uses the fitted average in the sample of countries analyzed as a benchmark.

Garg, Goyal and Pal [42] estimate the tax effort in 14 major Indian states using the stochastic frontier model. The principle of SFM used by the authors is the same as in Battese and Coelli [7] and Aigner, Lovell and Schmidt [37]. Garg, Goyal and Pal [42] found that tax capacity is influenced by the raise in tax base, but economic, demographic, infrastructural, political and fiscal incentive variables also play a significant role in the efficiency of collecting tax revenues.

Another interesting approach that uses the STFM approach is used in Alm and Duncan’s [43] study. These authors estimate the efficiency of a tax agency in collecting tax revenues. The empirical analysis uses Data Envelopment Analysis; for the second stage of the analysis, the authors use the SFM. The analysis is based on panel data for 34 OECD countries and 15 non-OECD countries for 2007–2011. Alm and Duncan [43] found that countries should be able to collect the current level of tax revenues using 10–15% less input at the tax agency level.

Langford and Ohlenburg [44] estimate the tax potential and tax effort for 85 non-resource-rich countries based on 27 years panel data. The authors use the industrial structure, education and trade as inputs for the STFM estimation and the main factors that influence the tax potential in the analyzed countries. Langford an Ohlenburg [44] define tax effort as the unused tax potential due to policy and enforcement factors. The authors use the stochastic tax frontier to estimate the maximum level of tax revenues that a country can collect using a set of inputs and environmental factors. According to Langford and Ohlenburg [44], the distinction between the set of inputs used and the exogenous factors is rendered by the degree and the distance of those factors from the immediate control of the government. Inputs directly affect the tax frontier (and thus tax capacity), whereas environmental factors affect inefficiency and represent those factors that cannot be controlled by the government. Three sets of inputs are regularly used by the STFM literature: structural economic factors and demographic and institutional factors.

Brun and Diakite [6] estimate the tax effort and tax potential for 114 countries and the effort and the potential of VAT in 57 countries using an unbalanced panel data analysis. The contribution of these authors to the literature stems from their particular focus on non-resource tax revenues, especially in countries that are natural- resource dependent and where the state draws a large share of its income from such resources. The authors build a comparative empirical analysis using a traditional regression analysis and a stochastic tax frontier model to estimate VAT and non-resource tax potential. Moreover, Brun and Diakite [6] use different types of SFM assumptions to compare the results obtained under time invariant technical inefficiency and time-varying technical inefficiency. The authors adopt the model proposed by Kumbhakar, Lien and Hardaker [8] to examine the differences between time-varying inefficiency and persistent inefficiency in collecting VAT revenues.

## Data

The data used for the empirical analysis, namely the VTTL and the VAT revenues, were taken from the studies of CASE [1, 2, 3, 4]. The analysis is based on a panel data for 26 European Union countries, including Austria, Belgium, Bulgaria, the Czech Republic, Denmark, Estonia, Finland, France, Germany, Greece, Hungary, Ireland, Italy, Latvia, Lithuania, Malta, the Netherlands, Poland, Portugal, Romania, Slovenia, Slovakia, Spain and the United Kingdom. The panel data are based on yearly observations for the period of 2000–2015. The data for Cyprus and Croatia are not available for the selected period, and therefore, we excluded these countries from the analyzed sample.

The exogenous variables, which are assumed to influence the inefficiency of VAT revenues, include the Corruption Perception Index (hereinafter CPI) obtained from the Transparency International Report for 2015; the shadow economy to GDP ratio obtained from Schneider [45, 46]; and economic openness. It is important to emphasize that previous studies, such as Brun and Diakite [6], rely on trading in percent of GDP as an indicator of economic openness. Using trading as an exogenous variable that influences VAT inefficiency can be questioned, considering that VAT in the EU is imposed only on imports and not imposed on exports (e.g., intracommunity trade is VAT exempted to avoid double taxation and exports to non-EU countries are zero-rated). Moreover, concerns regarding the endogeneity issue arise when using trade as an explanatory variable. This is because even if trade increases could lead to more VAT revenues, the government could use VAT to enhance trading. Therefore, we choose doing business variables as a proxy for economic openness. The data is obtained from the Doing Business Report from the Word Bank Database. We use the Trading Across Borders variables, selecting only import-related indicators, such as: documents needed to import (in number of documents required), time to import (expressed in days) and cost of import (expressed in US Dollars for each container). These exogenous variables report the time and cost of importing goods and services associated with three types of formalities: border compliance, documentary compliance and domestic transport of imported goods.

## Method

We adopt two different modeling strategies that belong to the second generation of stochastic frontier models: the inefficiency model of Battese and Coelli [7] and the latest innovation of SFM proposed by Kumbhakar, Lien and Hardaker [8] involving a model that allows separation between country effects, persistent inefficiency and time-varying inefficiency. The statistical analysis software used to estimate the STFM is STATA. We follow the study of Belotti et al. [47] where stochastic frontier analysis is adapted for STATA software. The authors describe the two main procedures used to estimate cross-sectional and panel data stochastic frontier models in STATA. Furthermore, Kumbhakar, Wang and Horncastle [48] comprehensively describe the data manipulation and technical efficiency estimation methods in stochastic frontier models for STATA. The first model adopted tackles the issues of heteroscedasticity associated with the inefficiency terms of the stochastic frontier approach, and the second model tackles the problem of heterogeneity among the analyzed countries.

The classic output-oriented stochastic production frontier model equation is as follows:

$$lny_{i}=f(x_{i};\beta )+\epsilon_{i}$$ (1)

$$\epsilon_{i}=v_{i}-u_{i};u_{i}\geq 0;$$ (2)

where *ϵ*_*i*_ represents the composed error term. The subcomponent *u*_*i*_ represents the log difference between the maximum output and the actual output. $u_{i}=lny_{i}^{*}-lny_{i}$, where *u*_*i*_*x*100% represents the percentage by which the actual level of tax revenues collected can be increased using the same level of inputs if the tax collection is fully efficient. *u*_*i*_ shows the amount of lost output due to technical inefficiency. Kumbhakar, Wang and Horncastle [48] specify that *y*_*i*_ represents the scalar of observed output, where the subscript denotes the observation for each country; *x*_*i*_ represents the *Jx*1 vector of input variables, *β* is the corresponding coefficient vector, *v*_*i*_ is a zero-mean random error term and *u*_*i*_ ≥ 0 is the production inefficiency. Once the set of input variables is determined, the SFM estimates the maximum level of output. Because *u*_*i*_ ≥ 0, the observed output is bounded below the frontier output level, $y_{i}^{*}$.

The common method used to estimate the SFM is to impose some sort of parameterization of distribution for the error components of *ϵ*_*i*_. This assumption reflects a parametric approach that imposes specific distributional assumptions on the error components *v*_*i*_ and *u*_*i*_ and applies a maximum likelihood method (hereinafter ML) to estimate technical inefficiency. Aigner, Lovell and Schmidt [37] and Meeusen and van de Broek [49] were the first economists to use a parametric approach where the composite error term is *ϵ*_*i*_ = *v*_*i*_ − *u*_*i*_. The choice of distributional assumption represents the core issue of the ML approach. Because *v*_*i*_ is considered as having a zero mean and a normal distribution, the *u*_*i*_ term or the inefficiency estimation depend on the type of distribution assumed (half-normal, truncated normal or exponential distribution). One of the most used distributions of *u*_*i*_ is the half-normal distribution, where the terms that compose the error term, *v*_*i*_ and *u*_*i*_, are assumed to be identically and independently distributed of each other and are expressed as $v_{i}∼i.i.d.,N(0,\sigma_{v}^{2})$ and $u_{i}∼i.i.d.,N^{+}(0,\sigma_{u}^{2})$.

According to Kumbhakar, Wang and Horncastle [48], the half-normal distribution has only one parameter and can capture various scenarios for each given variance. The half-normal distribution of *u*_*i*_ with low variance values tends to assume that the analyzed countries are clustered around zero and thus have a higher level of efficiency. When the value of the variance increases, the estimation of SFM finds highly efficient countries, and the resulting distributions will have a long tail. Because a half-normal distribution of *u*_*i*_ is assumed, wherein $u_{i}∼i.i.d.,N^{+}(0,\sigma_{u}^{2})$, the SFM yields the value of $\sigma_{u}^{2}$ (e.g., sigma u), which represents the shape of a half-normal distribution of *u*_*i*_ or the unconditional mean of *u*_*i*_. The unconditional mean represents the average technical inefficiency of the analyzed sample of countries. Kumbhakar, Wang and Horncastle [48] stress that if the objective of the research is to observe the technical inefficiency of each observation from the sample, then $\sigma_{u}^{2}$ is not a sufficient output to interpret. This issue has been solved by Jondrow et al. [50]. These authors propose a formula to calculate the conditional mean of *u*_*i*_ using the entire random error *ϵ*_*i*_, where *ϵ*_*i*_ = *v*_*i*_ − *u*_*i*_. *ϵ*_*i*_, or the composed error term contains information that is individual-specific, and the conditional mean of *u*_*i*_ is extracted to produce the observation-specific value of the inefficiency from the overall noise term.

The formula proposed by Jondrow et al. [50] to calculate the conditional mean of *u*_*i*_ is as follows:

$$E(u_{i}|\epsilon_{i})=\frac{\tau *\varphi (\frac{\mu_{*i}}{\sigma_{*i}})}{\Phi (\frac{\mu_{*i}}{\sigma_{*}})}+\mu_{*i}$$ (3)

Kumbhakar, Wang and Horncastle [48] conclude that the conditional mean of *u*_*i*_ estimated by SFM can be used to calculate the observation-specific estimate of technical efficiency using the formula in Eq (3). This method was first proved by Batese and Coelli [51], where *exp*(−*u*_*i*_) is given by the following:

$$E[\exp\left({-u}_{i}|\epsilon_{i}\right]=exp(-\mu_{*i}+\frac{1}{2}\sigma_{u}^{2})\frac{\Phi (\frac{\mu_{*i}}{\sigma_{*i}}-\sigma_{*})}{\Phi (\frac{\mu_{*i}}{\sigma_{*}})}$$ (4)

After *u*_*i*_ is predicted using the SFM approach, technical efficiency can be calculated, which represents another essential output of stochastic frontier model. The outputs of SFM produce the prediction of *u*_*i*_ and the level of technical efficiency (hereinafter TE) as follows:

$$TE=exp(-u_{i})$$ (5)

Greene [52] defines TE as the relationship between the observed output (i.e., VAT revenues) and some ideal or the optimum frontier estimated by the STFM. Since the common assumption of stochastic frontier models is that *u*_*i*,*t*_ ≥ 0, which represents a measure of technical inefficiency, and *TE* = exp(−*u*_*i*,*t*_), it follows that *TE* ≈ 0 < *TE* ≤ 1. Consequently, we assume that the VAT gap is the distance between the predicted technical efficiency of current VAT and the maximum amount of VAT estimated by the STFM. If EU countries were fully efficient in collecting VAT revenues and we assume no tax avoidance or tax evasion, then *TE = 1*. The estimated level of technical efficiency shows a clear picture of the tax efficiency and identifies those countries that underperform compared to countries that are very close to the efficiency frontier. The equation for calculating the VAT gap is as follows:

VATgap=1−TE (6)

The model adopted in this empirical analysis is based on a single input (i.e., VTTL) stochastic tax frontier. The stochastic tax frontier model is an output-oriented technical efficiency estimation, where *u*_*i*_ appears as an additional term that estimates the inefficiency or the inability of a country to collect the maximum level of tax revenues (i.e., VAT) for a given set of inputs (i.e., VTTL). The STFM also allows us to calculate the optimal frontier or the maximum amount of VAT that a country can collect given a set of inputs. Therefore, the sum *β*′*x* + *v*_*i*,*t*_ represents the optimal frontier, *β*′*x* represents the deterministic part of the STFM, and $v_{i,t}∼N(0,\sigma_{v}^{2})$ represents the stochastic part of the STFM. The sum *β*′*x* + *v*_*i*,*t*_ is the stochastic tax frontier. *u*_*i*,*t*_, or the inefficiency, is the amount by which countries fail to reach the optimum frontier in collecting VAT revenues.

The first model estimated in this paper follows the inefficiency model proposed by Battese and Coelli [7]. This model assumes that the inefficiency term *u*_*i*_ is a linear function of a set of exogenous explanatory variables. This approach is superior to the other modeling techniques of SFM because it takes into consideration the issue of heteroscedasticity. Greene [52] considers heteroscedasticity as a special case of heterogeneity that is concerned with the random parts of SFM and where shifts of inefficiency are explained by external factors that are not included in the output or input variables. The inputs are assumed to directly affect the tax frontier (and thus tax capacity), whereas the external factors affect inefficiency and are assumed to be outside government control. The single input inefficiency STFM equation is as follows:

$${lnVAT}_{i,t}=exp({lnVTTL}_{i,t}\beta +v_{i,t}-u_{i,t})$$ (7)

*lnVAT*_*i*,*t*_ represents the actual VAT revenues in million EUR in natural logarithm; *t* = 1,2…T for each *i-*th country, where *i* = 1,2, … N.

*lnVTTL* represents the VAT total tax liability in natural logarithm associated with *i*-th country at *t*-th observation, expressed in million EUR.

β is an unknown set of parameters to be estimated using the STFM approach based on Battese and Coelli’s [7] model. *v*_*i*,*t*_ is a random statistical error with i.i.d. $v_{i,t}∼N(0,\sigma_{v}^{2})$, and *u*_*i*,*t*_ are non-negative random variables associated with the technical inefficiency of VAT performance, with the distribution assumed to be truncated-normal where *u*_*i*,*t*_ is i.i.d., and $u_{i,t}∼N^{+}(\mu ,\sigma_{u}^{2})$ with a mean of *Z*_*i*,*t*_*δ* and a variance of *σ*^2^.

The inefficiency term *u*_*i*_ is obtained from the assumption it would be a function of explanatory variables *Z*_*i*,*t*_, where the following:

$$u_{i,t}=Z_{i,t}\delta +W_{i,t}$$ (8)

*Z*_*i*,*t*_*δ* represents a set of exogenous variables that affect the inefficiency of VAT performance. In our case, we include five exogenous variables: CPI, shadow economy as percent of GDP, documents needed to import (in number of documents required), time to import (expressed in days) and cost of import (expressed in US Dollars for each container). *W*_*i*,*t*_ is a set of random variables to be estimated; it is defined by truncation of the normal distribution of *u*_*i*,*t*_ with zero mean and a variance of $\sigma_{u}^{2}$. Our set of external factors (i.e., the *Z* variables) is selected after considering that these exogenous factors could influence the inefficiency of VAT revenues.

The CPI is expected to decrease the inefficiency term *u*_*i*,*t*_. More precisely, an increase in the CPI decreases the level of corruption and thereby decreases the inefficiency of VAT. The shadow economy has a positive impact on technical inefficiency, since it represents the legal and illegal undeclared economic activities that have a direct negative impact on tax revenues and therefore increases inefficiency. The impact of documents needed to import is expected to increase VAT inefficiency as well as the time to import. Cost of import is expected to decrease VAT inefficiency.

The second model adopted to estimate the STFM that addresses heterogeneity issues in this paper has been proposed by Kumbhakar, Lien and Hardaker [8]. This model allows the separation of country effects, persistent inefficiency and time-varying inefficiency. Kumbhakar, Wang and Horncastle [48] highlight a common limitation of the first generation of SFM represented by a lack of separation between individual heterogeneity and time-persistent inefficiency. The authors follow the recommendation previously made by Mundlak [53], which underlines the importance of disentangling time-varying and persistent inefficiency to observe the country-specific, time-independent effects of inputs on efficiency. The country-specific inefficiency or the persistent inefficiency depends only on government decisions concerning fiscal policy.

Kumbhakar, Lien and Hardaker [8] and Kumbhakar, Wang and Horncastle [48] propose a further decomposition of the error term *ϵ*_*i*_ into four components. This latest approach intends to separate country effects from persistent inefficiency, because the two subcomponents of *u*_*i*_ tend to confound country effects with persistent inefficiency. Kumbhakar, Wang and Horncastle [48] emphasized that this treatment of country heterogeneity that is included into persistent inefficiency could produce upwardly biased inefficiency estimates. Thus, the STFM equation becomes the following:

$$ln{VAT}_{i,t}=\alpha_{0}+{lnVTTL}_{i,t}^{\prime }\beta +\mu_{i,t}+v_{i,t}-\eta_{i}{-u}_{i,t}$$ (9)

The first component of *ϵ*_*i*_, namely *μ*_*i*,*t*_, captures the country’s latent heterogeneity that needs to be separated from the inefficiency term, as stated by Kumbhakar, Wang and Horncastle [48]; *v*_*i*,*t*_ represents the standard statistical white noise; the third subcomponent, *η*_*i*_, represents the persistent country-specific inefficiency; and the fourth subcomponent, *u*_*i*,*t*_, is the random, time-varying technical inefficiency.

To estimate these four subcomponents of *ϵ*_*i*_ using panel data based on the STFM, Kumbhakar, Wang and Horncastle [48] recommend a three-step procedure. The equation of the STFM three-step procedure is as follows:

$$ln{VAT}_{i,t}=\alpha_{0}^{*}+f(lnVTTL;\beta )+\alpha_{i}+\epsilon_{i}$$ (10)

Where $\alpha_{0}^{*}=\alpha_{0}-E(\eta_{i})-E(u_{i,t})$; *α*_*i*_ = *μ*_*i*_ − *η*_*i*_ + *E*(*η*_*i*_); and *ϵ*_*i*_ = *v*_*i*,*t*_ − *u*_*i*,*t*_ + *E*(*u*_*i*,*t*_).

The assumed distributions of the four error subcomponents are as follows: $v_{i}∼i.i.d.,N(0,\sigma_{v}^{2})$; $u_{i}∼i.i.d.,N^{+}(0,\sigma_{u}^{2});\;\mu_{i}∼i.i.d.,N(0,\sigma_{\mu }^{2})$; and $\eta_{i}∼i.i.d.,N^{+}(0,\sigma_{\eta }^{2})$. Since the second and third steps make use of standard stochastic frontier analysis, we can assume that the distribution of *u*_*i*_ and *η*_*i*_ is exponential; that is, $u_{i}\approx exp(\sigma_{u}^{2})$, and $\mu_{i}\approx exp(\sigma_{\mu }^{2})$. The choice of distribution (i.e., exponential or half-normal) for the inefficiency term depends on the value of the log-likelihood.

In the first step, we use a random effect standard Generalized Least Squares (hereinafter GLS) panel data regression to estimate $\hat{\beta }$, as in Kumbhakar, Lien and Hardaker [8], Kumbhakar, Wang and Horncastle [48] and Brun and Diakite [6]. In this step we obtain the estimated values of country effects *α*_*i*_ and the noise term *ϵ*_*i*_, denoted by $\hat{\alpha_{i}}$ and $\hat{\epsilon_{i,t}}$, respectively.

In the second step, the time-varying technical inefficiency *u*_*i*,*t*_ is predicted by using the *ϵ*_*i*_ estimates obtained in the first step. The estimation of *u*_*i*,*t*_ is done using a standard stochastic frontier technique as recommended by Kumbhakar, Wang and Horncastle [48]. In this second step, we can predict the residual technical inefficiency component, $\hat{u_{i,t},}$ using the formula proposed by Jondrow et al. [50]. Time-varying technical efficiency (hereinafter TTE) is calculated as *TTE* = exp(−*u*_*i*,*t*_). The time-varying VAT gap is calculated using the formula from Eq (6).

In the third step, we estimate the persistent technical inefficiency *η*_*i*_ by applying the same standard stochastic frontier procedure adopted in the second step. To predict *η*_*i*_, we use the *α*_*i*_ estimates obtained in the first step. Persistent technical efficiency (hereinafter PTE) is calculated as *PTE* = exp(−*η*_*i*_). The persistent VAT gap is calculated using the formula from Eq (6). The overall technical efficiency (hereinafter OTE) is calculated as *OTE* = *PTE x TTE*, as in Kumbhakar, Wang and Horncastle [48] and Brun and Diakite [6]. The overall VAT gap is calculated using the formula from Eq (6).

## Results

Before estimating the STFM using the models described in the previous section, we follow the recommendation of Schmidt and Lin [54] that stresses that a test of the OLS residuals should be performed to check the validity of the stochastic frontier specification. Because the STFM is an output-oriented model and pertains to the production-type stochastic frontier models, the composed the error term *ϵ*_*i*,*t*_ = *v*_*i*,*t*_ − *u*_*i*,*t*_, where *u*_*i*,*t*_ ≥ 0 and *v*_*i*,*t*_, is distributed symmetrically around zero. Kumbhakar, Wang and Horncastle [48] note that the residuals predicted from a standard OLS estimation should be skewed to the left, that is, the error term should have a negative skew. We estimate a standard GLS regression where the dependent variable is VAT (*lnVAT*) expressed in natural logarithm, and the independent variable is VTTL (*lnVTTL*) in natural logarithm; we predict the error term to analyze the presence of negative skewness.

As shown in Table 1, the error term *ϵ*_*i*,*t*_ has a skewness of -1.040, which is consistent with stochastic tax frontier model specification. The summary statistics are presented in Table A.1 in S1 Appendix.

**Table 1:** Skewness test of the statistical noise.

*e*_*i*,*t*_				
	Percentiles	Smallest		
1%	-0.2531291	-0.3108		
5%	-0.1124332	-0.28515		
10%	-0.067834	-0.27648	Obs	416
25%	-0.024815	-0.25713	Sum of Wgt.	416
50%	0.0041211		Mean	2.46E-11
		Largest	Std. Dev.	0.065413
75%	0.032561	0.166075		
90%	0.0675052	0.170518	Variance	0.004279
95%	0.0996195	0.171646	**Skewness**	**-1.04001**
99%	0.1613496	0.172305	Kurtosis	7.043878

Source: Own calculation.

The first STFM estimated follows the approach of Battese and Coelli’s [7] time-varying inefficiency model. This model takes into account the issue of heteroscedasticity by assuming that the distribution of inefficiency is also affected by other external factors rather than merely the inputs of the stochastic frontier model. This model combines both estimations–the STFM and the inefficiency model–in one step using a maximum likelihood method. A two-step approach can lead to biased estimates in efficiency analysis, as pointed by Hang and Schimdt [55]. The distribution of *u*_*i*,*t*_ is assumed to be truncated-normal, where $u_{i,t}∼N^{+}(\mu ,\sigma_{u}^{2})$ with mean of *Z*_*i*,*t*_*δ* and a variance of *σ*^2^. The distribution of the inefficiency term is assumed to be a linear function of CPI, shadow economy, documents needed to import, time to import and cost of import. In this model we parametrize the pre-truncated mean of inefficiency—*μ* (Mu), or the pre-truncated mean, and the variance of *u*_*i*,*t*_ of the inefficiency distribution, by the aforementioned external factors.

Table 2 shows the results of Battese and Coelli’s [7] STFM estimation, where the dependent variable is VAT (*lnVAT*) and the explanatory variable is VTTL (*lnVTTL*). This is an output-oriented STFM with only one input. The second part of Table 2 shows the inefficiency model, where the pre-truncated mean Mu (*μ*) of the distribution of *u*_*i*,*t*_ and the variance of *u*_*i*,*t*_ is parametrized as a function of CPI, shadow economy, documents needed to import, time to import and cost of import. In the case of mean pre-truncated inefficiency, we found that four out of five exogenous factors are statistically significant and have the expected impact on the technical inefficiency of VAT. sigma_u ($\sigma_{u}^{2}$) represents the variance of the inefficiency term, *u*_*i*,*t*_; sigma_v ($\sigma_{v}^{2}$) is the variance of the statistical noise, *v*_*i*,*t*_; and lambda (*λ*) provides information regarding the relative contribution of *u*_*i*,*t*_ to the variation of the total error term, where lambda is equal to the following:

$$\lambda =\sigma_{u}/\sigma_{v}$$ (11)

**Table 2:** Stochastic tax frontier model estimation, panel data for 26 EU countries, 2000–2015.

Dependent variable lnVAT	Stochastic Tax Frontier Model (truncated-normal)	
lnVTTL	0.988***	(0.00274)
_cons	0.0611*	(0.0294)
Inefficiency Model		
Mu (μ)		
CPI	-0.465****	(0.0987)
Shadow economy	0.618***	(0.141)
Documents to import	0.328***	(0.0811)
Time to import	-0.0661	(0.0506)
Cost to import	-0.260**	(0.0982)
_cons	1.408	(0.884)
Usigma		
CPI	-2.583***	(0.688)
Shadow economy	-2.409***	(0.370)
Documents to import	-0.682*	(0.272)
Time to import	0.705*	(0.333)
Cost to import	-0.227	(0.372)
_cons	14.86**	(5.617)
Vsigma		
_cons	-6.775***	(0.165)
*N*	416	
Log Likelihood	509.1	
sigma_u ($\sigma_{u}^{2}$)	0. 163	(0.00835)
sigma_v ($\sigma_{v}^{2}$)	0.0337	(0.00278)
lambda (λ)	4.9394	(0.00925)

Source: Own calculation; Standard errors in parentheses

* p < 0.05

** p < 0.01

*** p < 0.001.

Kumbhakar, Wang and Horncastle [48] emphasize that the interpretation of the impact of exogenous determinants on inefficiency should be treated cautiously, since the maximum likelihood estimates of Mu (*μ*) are not very informative. This dynamic exists because the relationship between E (*u*_*i*_) and the external factors (*Z*_*i*,*t*_) is not linear, and the slope coefficient of *W*_*u*_ does not represent the marginal effect of the *Z*_*i*,*t*_ variables. At first glance, the inefficiency model in Table 2 offers one useful output, which is the sign of the estimated coefficients. The sign indicates the impact of external factors on the technical inefficiency of VAT. The Corruption Perception Index (CPI) has a negative impact on technical inefficiency. An increase in CPI, which reflects a decrease in corruption, reduces the inefficiency of VAT collection. The shadow economy has an opposite effect; an increase in the shadow economy increases the inefficiency of VAT. The number of documents needed to import goods and services has a positive impact on inefficiency. This means that as bureaucracy increases, tax compliance is negatively affected. We found that time to import is not statistically significant in the case of the inefficiency model. The last import-related indicator, the cost to import, plays a negative role on VAT inefficiency. This result shows that inefficiency in collecting the VAT revenues tends to decrease as the value of imports increases. This intriguing estimate reveals the fact that small-value imports tend to be underreported in order to avoid VAT, and as the value of imports increases, taxpayers become more tax compliant.

To capture the magnitude of the *Z*_*i*,*t*_ variables on technical inefficiency, *u*_*i*,*t*_, Kumbhakar, Wang and Horncastle [48] recommend a calculation of marginal effects.

Table 3 shows the marginal effects of each external determinant on technical inefficiency. The highest magnitude of CPI is found in the eastern EU countries. The decrease in corruption has a significant negative impact on VAT inefficiency, especially in Bulgaria, Romania, Slovakia, Latvia, Lithuania, Hungary, the Czech Republic, Estonia and Poland. For more details, see Graph A in S2 Appendix. In the case of southern EU countries, CPI plays an important role, particularly in Greece, Italy and Malta. CPI has a small impact on VAT inefficiency in western EU countries, but it has a negative effect on technical inefficiency. For details, see Graph B in S2 Appendix. The marginal effects of the shadow economy follow the same pattern as CPI, where the eastern EU countries seem to be the most affected, particularly Bulgaria, Romania, Slovakia, Latvia, Lithuania, Hungary, the Czech Republic, Estonia and Poland. The shadow economy tends to increase VAT inefficiency in southern EU countries, particularly in Greece, Malta and Italy. Regarding the western EU group of countries, the shadow economy has a considerably low impact and a positive marginal effect on VAT inefficiency. The number of documents required to import goods and services tend to increase the VAT inefficiency significantly in eastern EU countries. Bureaucracy tends to increase VAT noncompliance, especially in case of Romania, Lithuania, Bulgaria, Estonia and, to a lesser extent, the rest of the eastern group of EU countries. For southern EU countries, documentary compliance plays a highly negative role on VAT compliance in Malta and Greece. The number of documents needed to import has a significantly lower impact on VAT inefficiency in western EU countries. The cost of imports has a negative impact on VAT inefficiency in case of eastern EU countries. Small-value imports tend to go unreported, especially in Romania, Bulgaria, Estonia, Lithuania, Latvia and Poland. In southern EU countries, the value of imports has also a negative impact on VAT inefficiency; the highest estimates were obtained for Malta and Greece. In case of western EU countries, the cost to import has a negative impact on the inefficiency of VAT, but this is a smaller amount when compared to the aforementioned EU countries. For more details, see Graph C in S2 Appendix.

**Table 3:** The marginal effects of the CPI, shadow economy, documents to import and cost to import on the mean *E(u)* of the inefficiency term *u*_*i*,*t*_.

Country	Marginal effect of CPI on E(u)	Marginal effect of shadow economy on E(u)	Marginal effect of documents to import on E(u)	Marginal effect of cost to import on E(u)
Austria	-0.20965	-0.11967	-0.02287	-0.03417
Belgium	-0.10162	-0.02462	0.003217	-0.02351
Bulgaria	-0.46367	0.497469	0.275955	-0.2341
Czech Republic	-0.43637	-0.02934	0.046542	-0.11683
Denmark	-0.06832	-0.02479	-0.00137	-0.01409
Estonia	-0.43206	0.52294	0.282587	-0.23049
Finland	-0.11942	-0.01335	0.010458	-0.03087
France	-0.22614	-0.07868	-0.00308	-0.04734
Germany	-0.12476	-0.03793	0.000649	-0.02726
Greece	-0.45783	0.281467	0.182616	-0.18752
Hungary	-0.39489	0.17873	0.130072	-0.14843
Ireland	-0.16674	-0.08907	-0.01558	-0.02845
Italy	-0.35084	-0.05734	0.022959	-0.08691
Latvia	-0.44875	0.442218	0.250261	-0.2184
Lithuania	-0.45475	0.538211	0.292203	-0.24006
Luxembourg	-0.1275	-0.07404	-0.01445	-0.02052
Malta	-0.45715	0.573033	0.307448	-0.24798
Netherlands	-0.10341	-0.04807	-0.00659	-0.01913
Poland	-0.39879	0.149135	0.117919	-0.14337
Portugal	-0.24023	-0.01391	0.026581	-0.06478
Romania	-0.46435	0.531178	0.29049	-0.2413
Slovakia	-0.42466	0.064255	0.085056	-0.133
Slovenia	-0.33441	0.138362	0.104583	-0.12299
Spain	-0.17756	-0.03321	0.009822	-0.04311
Sweden	-0.07713	-0.02527	-0.00038	-0.01647
United Kingdom	-0.12199	-0.06116	-0.00968	-0.02165
EU-26 average	-0.28396	0.12256	0.090978	-0.10549

Source: Own calculation.

After the STFM estimation, the next step is to estimate technical efficiency using the formula proposed by Jondrow et al. [50], which is presented in Eq (5). Once the size of TE is obtained, we apply the formula from Eq (6) to calculate the VAT gap.

Table 4 presents the estimation of VAT gap in the EU-26 countries as well as the comparative results of CASE [1, 2, 3, 4] reports. The first column presents the average VAT gap obtained by CASE [1, 2, 3, 4], and the second column presents the estimation of the VAT gap using the model of Battese and Coelli [7]. It is important to emphasize that the estimated VAT gap is presented in terms of percentage of the optimal frontier or some ideal level of VAT revenues that would have been collected under full tax compliance.

**Table 4:** Results from panel data STFM estimation of VAT gap using Battese and Coelli [7] model for EU-26 countries, 2000–2015.

Country	CASE VAT gap average 2000–2015 in % of VTTL	VAT Gap by STFM in %	Standard Error	[95% Confidence Interval]	
Austria	10.23	4.19	0.273296	3.609907	4.774942
Belgium	12.48	4.31	0.299902	3.667053	4.945506
Bulgaria	18.66	16.32	1.935624	12.19416	20.44554
Czech Republic	20.96	16.44	1.707507	12.80335	20.08228
Denmark	10.08	2.34	0.121959	2.082884	2.602782
Estonia	13.44	11.73	1.091735	9.403974	14.05793
Finland	10.33	4.05	0.50028	2.981164	5.113807
France	13.39	5.37	0.577202	4.144505	6.605058
Germany	11.73	3.16	0.197403	2.735365	3.576875
Greece	28.83	24.25	0.939672	22.24809	26.25382
Hungary	23.48	18.97	1.068065	16.69568	21.24873
Ireland	9.27	4.59	0.717625	3.059814	6.118976
Italy	26.76	19.74	0.715309	18.21266	21.26195
Latvia	22.44	20.26	2.074112	15.8423	24.68404
Lithuania	32.69	29.47	1.212078	26.8884	32.05537
Luxembourg	8.47	4.64	0.651268	3.252028	6.028318
Malta	22.85	21.52	2.884073	15.37238	27.66689
Netherlands	6.35	1.89	0.183768	1.497413	2.280798
Poland	17.13	12.06	1.530514	8.794468	15.3189
Portugal	9.66	5.22	0.896163	3.312511	7.132764
Romania	40.28	36.68	1.168788	34.19038	39.1728
Slovakia	28.97	25.25	1.170455	22.75103	27.74056
Slovenia	6.50	4.13	0.256077	3.58918	4.680812
Spain	9.99	4.87	1.700627	1.241719	8.491322
Sweden	3.52	1.17	0.042735	1.080157	1.262331
United Kingdom	11.59	3.17	0.228651	2.680851	3.655567
EU-26 average	16.54	11.76	0.9286495	9.781977	13.740718

Source: Own calculation and CASE [1, 2, 3, 4].

The most efficient country in terms of VAT revenues is Sweden with a VAT gap of only 1.17% compared to CASE results of 3.52% of VTTL. At the opposite end stands Romania, which has the highest STFM-estimated VAT gap of 36.68%, still smaller than the CASE average of 40.28% of VTTL. The other less efficient EU countries that have a high VAT gap are Lithuania with 29.47%, Slovakia with 25.25%, Greece with 24.25%, Malta with 21.52%, Latvia with 20.26% and Italy with 19.74%. The western group of EU countries proves to be more efficient in terms of VAT gap; the smallest averages are had in the Netherlands with 1.89%, Denmark with 2.34%, Germany with 3.16%, Austria with 4.19% and Finland with 4.05%. From the eastern group of EU countries, the smallest STFM-estimated VAT gap is in Slovenia, with only 4.13% of the optimal frontier. The results under the STFM appear to be significantly lower than the results obtained through the top-down method used by the CASE reports. The only close match regarding the size of VAT gap between the STFM and a top-down method is Malta. Overall, there is a difference of more than 5% between the average VAT gap for the 26 EU countries obtained by the STFM and those reported by CASE reports.

Following the debate mentioned in Kumbhakar, Wang and Horncastle [48], there is a fair amount of doubt regarding the accuracy of TE estimated through the STFM based on Battese and Coelli [7]. This doubt exists because the model of Battese and Coelli does not separate between country heterogeneity and inefficiency, which could in turn lead to biased estimates. However, one important advantage of Battese and Coelli’s [7] model is the assessment of heteroscedasticity and the prediction of the impact of external factors on VAT technical inefficiency, which could prove relevant to policy.

The latest innovation in stochastic frontier models attempts to overcome the unaddressed issues in previous models. We choose the STFM model that addresses the heterogeneity issues by allowing the separation of country effects, persistent inefficiency and time-varying inefficiency; this model was proposed by Kumbhakar, Lien and Hardaker [8] and later expanded by Kumbhakar, Wang and Horncastle [48]. This is a three-step model that decomposes the error term in four subcomponents, allowing the STFM estimation of time-varying inefficiency and persistent inefficiency. Therefore, we can estimate time-varying and persistent TE and the corresponding time-varying and persistent VAT gap.

In the first step of the analysis, we run a standard GLS regression with random effects from which we predict the size of country effects *α*_*i*_ and the noise effects *ϵ*_*i*_; the dependent variable is VAT, the explanatory variable is VTTL, and both are expressed in natural logarithms. In the second step, we run a standard stochastic frontier model to estimate time-varying VAT inefficiency using the estimates of country effects *α*_*i*_ obtained in the first step. In the third step, we repeat the procedure to estimate the persistent VAT inefficiency using the estimates of *ϵ*_*i*_ obtained in the first step. As stated in the previous section, we run the second and third steps assuming both half-normal distribution and exponential distribution of the inefficiency terms. We choose the latter because the obtained value of log-likelihood is higher for the exponential distribution than for the half-normal distribution, which represents a goodness-of-fit test.

Table 5 shows the estimation results for time-varying and persistent VAT inefficiency under the STFM based on Kumbhakar, Lien and Hardaker’s [8] recommended procedure.

**Table 5:** The estimation of time-varying and persistent VAT inefficiency by the stochastic tax frontier model for EU-26 countries, 2000–2015.

	Time-varying inefficiency (Exponential)		Persistent inefficiency (Exponential)	
Dep. Variable	*ϵ*_*i*_		*α*_*i*_	
Constant^1^	0.0435***	(0.00411)	0.123***	(0.00322)
Usigma				
_cons	-6.272***	(0.187)	-4.186***	(0.109)
Vsigma				
_cons	-6.199***	(0.110)	-7.398***	(0.173)
*N*	416		416	
Log Likelihood	572.5		381.0	
sigma_u ($\sigma_{u}^{2}$)	0.0435		0.123	
sigma_v ($\sigma_{v}^{2}$)	0.0451		0.0247	
Lambda (λ)	0.964		4.984	

Source: Own calculation; Standard errors in parentheses

* *p* < 0.05

** *p* < 0.01

*** *p* < 0.001

^1^ a new variable was created, named *constant* = 1, because a constant is needed in order to force the standard stochastic procedure in STATA, following the example recommended by Kumbhakar, Wang and Horncastle [48].

The first column of Table 5 presents the time-varying VAT inefficiency. As seen in the table, the time-varying inefficiency is low compared with the persistent inefficiency. The lambda value (λ = 0.964) shows that the amount of variance in the total error term due to the inefficiency term is relatively small. The second column presents the estimated persistent inefficiency of VAT. The value of lambda is high (λ = 4.984), which means that the variation of total error term is largely due to the inefficiency term. Both time-varying and persistent inefficiency terms are statistically significant.

After the estimation of the three-step STFM using the model of Kumbhakar, Lien and Hardaker [8], we calculate the time-varying technical efficiency and persistent technical efficiency of the VAT using the formula proposed by Jondrow et al. [50], which is presented in Eq (5). The predicted levels of inefficiency include also confidence intervals where the lower bound and upper bound of inefficiency are also predicted. The median, lower bound and upper bound of time-varying and persistent technical efficiency calculated levels are presented in Table A.2 and Table A.3 in S1 Appendix. Additionally, we calculate the overall technical efficiency of VAT, where OTE = TTE x PTE. Because the central objective of this study is to determine the VAT gap in the EU, we also calculate the time-varying VAT gap, the persistent VAT gap and the overall VAT gap using the formula in Eq (6). The median, lower bound and upper bound of overall technical efficiency as well as time varying, persistent and overall VAT gap are also presented in Table A.4, Table A.5, Table A.6 and Table A.7 in S1 Appendix.

Table 6 shows the obtained results regarding the time-varying technical efficiency of VAT, the time-varying VAT gap, the persistent technical efficiency of VAT and the persistent VAT gap, the overall technical efficiency of VAT and the overall VAT gap. For comparison reasons, we also added a seventh column showing the VAT gap averages obtained from CASE [1, 2, 3, 4] reports. On average, the time-varying VAT gap in the 26 EU countries is low, amounting to only 4.19% of the optimum frontier estimated by the STFM. The average persistent VAT gap is considerably higher for the 26 EU countries, amounting to 11.06% of the optimum frontier. The size of the overall VAT gap obtained under the STFM and the VAT gap presented in CASE reports using a top-down method are notably different. Our estimation tends to be lower than the benchmark results of CASE [4]; the average overall VAT gap in the 26 EU countries is 14.73% in our estimate compared to an average VAT gap of 16.54% of VTTL obtained by CASE reports. However, observing each country separately, we do find close matching between the STFM and the top-down method, such as in the case of Bulgaria, the Czech Republic, Estonia, Hungary, Latvia, Lithuania and Slovenia, where the estimates of the two methods differ by no more than 1%.

**Table 6:** Results of the STFM estimation of time-varying and persistent technical efficiency VAT and VAT gap using the model of Kumbhakar, Lien and Hardaker [8] for EU-26, 2000–2015.

Country	Time-varying efficiency of VAT in %	Time-varying VAT gap in %	Persistent efficiency of VAT in %	Persistent VAT gap in %	Overall Technical Efficiency of VAT in %	Overall VAT gap in %	CASE VAT gap average 2000–2015
Austria	96.56	3.44	96.21	3.79	92.89	7.11	10.23
Belgium	96.53	3.47	94.23	5.77	90.96	9.04	12.48
Bulgaria	94.95	5.05	86.05	13.95	81.70	18.30	18.66
Czech Republic	95.21	4.79	84.50	15.50	80.45	19.55	20.96
Denmark	96.62	3.38	96.34	3.66	93.09	6.91	10.08
Estonia	96.01	3.99	91.10	8.90	87.46	12.54	13.44
Finland	96.29	3.71	95.88	4.12	92.33	7.67	10.33
France	96.40	3.60	94.45	5.55	91.05	8.95	13.39
Germany	96.57	3.43	96.13	3.87	92.84	7.16	11.73
Greece	95.94	4.06	76.73	23.27	73.61	26.39	28.83
Hungary	95.98	4.02	81.97	18.03	78.68	21.32	23.48
Ireland	96.15	3.85	96.51	3.49	92.79	7.21	9.27
Italy	96.25	3.75	80.08	19.92	77.08	22.92	26.76
Latvia	94.59	5.41	81.56	18.44	77.14	22.86	22.44
Lithuania	95.34	4.66	71.54	28.46	68.20	31.80	32.69
Luxembourg	96.16	3.84	96.29	3.71	92.60	7.40	8.47
Malta	92.15	7.85	79.86	20.14	73.59	26.41	22.85
Netherlands	96.47	3.53	98.38	1.62	94.91	5.09	6.35
Poland	95.27	4.73	89.08	10.92	84.86	15.14	17.13
Portugal	96.02	3.98	96.29	3.71	92.46	7.54	9.66
Romania	95.18	4.82	64.31	35.69	61.21	38.79	40.28
Slovakia	95.59	4.41	75.81	24.19	72.47	27.53	28.97
Slovenia	96.56	3.44	97.61	2.39	94.26	5.74	6.50
Spain	95.22	4.78	96.57	3.43	91.95	8.05	9.99
Sweden	96.59	3.41	98.93	1.07	95.56	4.44	3.52
United Kingdom	96.57	3.43	96.09	3.91	92.80	7.20	11.59
EU-26 average	95.81	4.19	88.94	11.06	85.27	14.73	16.54

Source: Own calculation and the reports of CASE [1, 2, 3, 4].

Comparing the results obtained from the STFM estimation of VAT gap and the estimates of HRMC [14], we found that the UK had an average 7.2% VAT gap between 2000–2015, while HMRC [14] estimated an average VAT gap of 9.95% between 2005–2015. The 2.75% difference can be justified by a shorter period being analyzed by HMRC [14]. Moreover, in order to better assess the differences between the HMRC [14] estimates of VAT gap and our STFM estimates, we chose to present the evolution of VAT gap estimates produced by HMRC from 2011 until 2018.

In Table 7 we show the estimates of VAT gap calculated by HRMC from 2011 to 2018. HRMC includes the 2004 tax year only in 2011 and 2012, while the following calculations start with the 2005 tax year. As pointed out in the second section of this paper, HMRC [14] underlines several limitations with their tax gap estimates, and they also mention that every year the British tax agency revises and recalculates the tax gap levels for past years. The constant revision of VAT gap for past years is due to the updated data and methodological improvements according to HMRC [14]. When observing the evolution of VAT gap estimates and the yearly revision, it is important to mention that the VAT gap levels revised by HMRC do not have large differences between 2011–2015. However, the VAT gap recalculation from 2016 onwards shows large differences in VAT gap levels in years past. The constant revision of VAT gap levels and tax gap estimation based partially on forecasts by the HMRC raises questions regarding the accuracy of their tax gap estimates. The overall image shows a clear downward trend in VAT gap in the UK; the revised size of VAT gap is decreasing each year. Therefore, we consider the STFM estimates of VAT gap for the UK to be more accurate when taking into consideration the revised VAT gap estimates that show that the VAT gap was lower than initially estimated.

**Table 7:** The changes in VAT gap estimates from 2011 to 2018 calculated by HM revenues & customs in the United Kingdom.

Tax year	HMRC (2011)	HMRC (2012)	HMRC (2013)	HMRC (2014)	HMRC (2015)	HMRC (2016)	HMRC (2017)	HMRC (2018)
2004–05	11.70%	9.80%	n.a.	n.a.	n.a.	n.a.	n.a.	n.a.
2005–06	15.20%	13.10%	14.40%	14.40%	14.70%	14.50%	13.64%	12.53%
2006–07	13.50%	11.60%	12.90%	12.90%	12.90%	12.70%	11.80%	10.81%
2007–08	12.40%	10.50%	11.70%	11.70%	11.70%	11.40%	10.50%	9.16%
2008–09	15.50%	12.40%	14.20%	14.20%	14.70%	14.50%	13.70%	12.55%
2009–10	13.80%	10.80%	11.60%	11.60%	12.60%	12.20%	11.30%	10.34%
2010–11	n.a.	10.10%	10.40%	10.30%	11.20%	10.60%	9.75%	8.85%
2011–12	n.a.	n.a.	10.40%	10.40%	11.70%	11.30%	10.45%	9.29%
2012–13	n.a.	n.a.	n.a.	10.90%	11.90%	11.80%	11.06%	9.50%
2013–14	n.a.	n.a.	n.a.	n.a.	11.10%	11.40%	11.42%	10.02%
2014–15	n.a.	n.a.	n.a.	n.a.	n.a.	10.30%	10.00%	9.04%
2015–16	n.a.	n.a.	n.a.	n.a.	n.a.	n.a.	9.81%	8.44%
2106–17	n.a.	n.a.	n.a.	n.a.	n.a.	n.a.	n.a.	8.91%
**Average**	**13.68%**	**11.19%**	**12.23%**	**12.05%**	**12.50%**	**12.07%**	**11.22%**	**9.95%**

Source: HRMC [14].

As emphasized by Kumbhakar, Wang and Horncastle [48], the model that separates country heterogeneity from time-varying inefficiency and disentangles the time-varying inefficiency from persistent inefficiency offers a clear advantage compared to the previous SFMs. From a policy recommendation standpoint, it is more relevant to determine the size of time-varying VAT gap and persistent VAT gap because the required corrective measures to address the country-specific VAT gap differ considerably from the time-varying VAT gap. A detailed description regarding the potential causes of time-varying and persistent VAT inefficiency is provided in Brun and Diakite [6]. Since time-varying VAT inefficiency is country- and time-dependent, Brun and Diakite [6] consider that it could be related to the experience of tax officers and their performance in collecting VAT revenues. Persistent VAT inefficiency is strictly country-specific and is related to each country’s tax law, the form of organization of revenue services and the organization of municipal tax administrations. In addition, persistent VAT inefficiency depends on each country’s tax morale, culture and fiscal policy. To provide better comprehension, we chose to graphically present the time-varying and persistent VAT gap by dividing the 26 EU countries into three distinct groups: the western group, the southern group and the eastern group.

**Fig 1:**
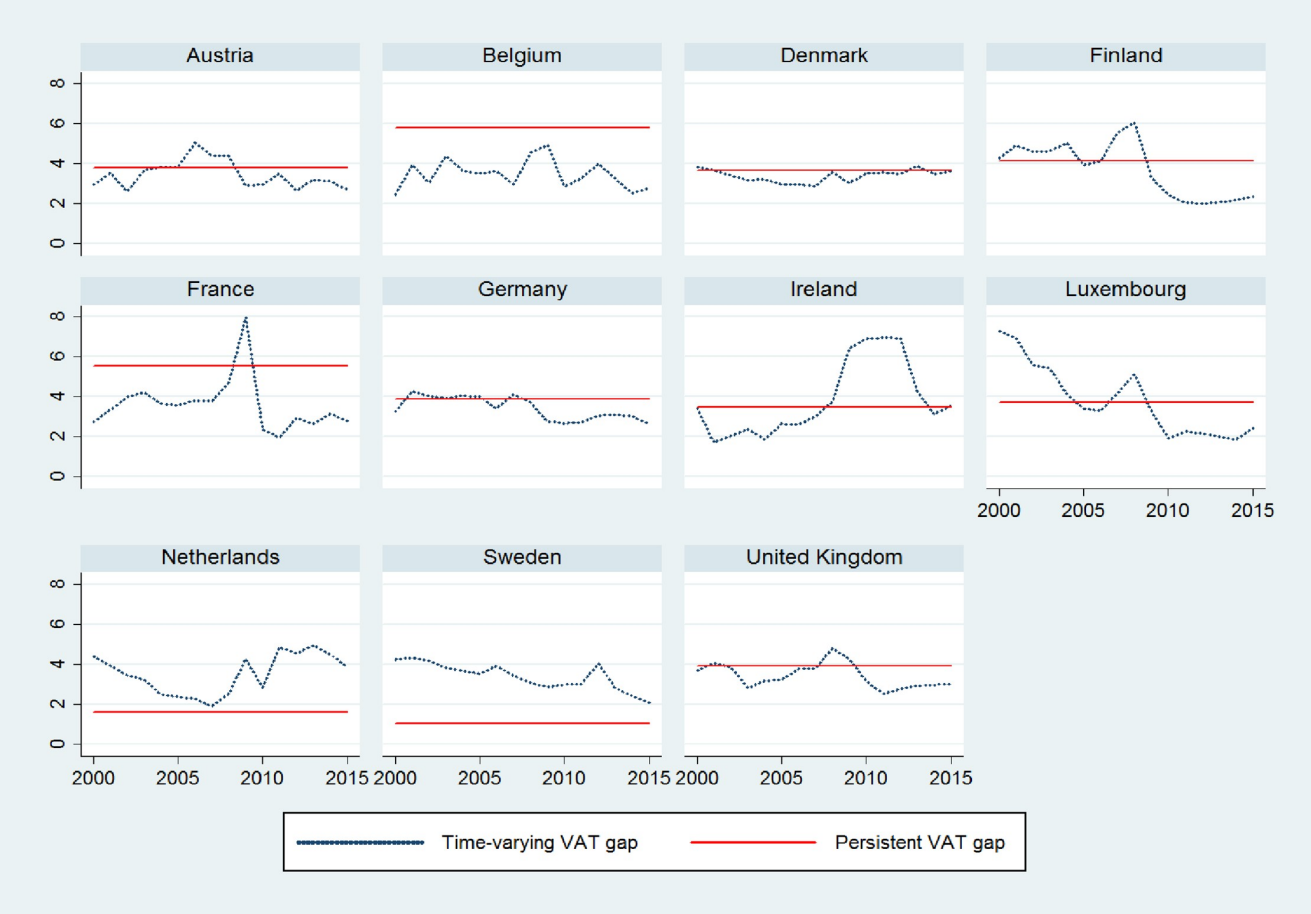
Time-varying VAT gap and persistent VAT gap in the western group of EU countries. The blue dotted line represents the time-dependent Value Added Tax gap and the red line represents the country-specific or persistent Value Added Tax gap, between 2005–2015. The persistent and time-dependent VAT gap was generated in STATA after the STFM estimation.

Fig 1 presents the differences between the time-varying and persistent VAT gap. This graph shows that Sweden, the Netherlands and Finland exhibit a larger time-varying VAT gap than the persistent VAT gap.

This finding means that the experience and performance of tax agents matters more than tax law or tax morale for VAT efficiency. A different pattern is observed in Austria, Germany, Denmark, Ireland and the United Kingdom, where the contribution of tax agents’ experience or performance and the country-specific tax law or tax morale play almost an equal role in VAT efficiency. In the case of France, Belgium and Luxembourg, the persistent VAT gap tends to be higher than the time-varying VAT gap, which shows that the inefficiency is more affected by country-specific tax morale, the organization of tax administrations and issues related to tax law than by the experience and performance of tax agents.

**Fig 2:**
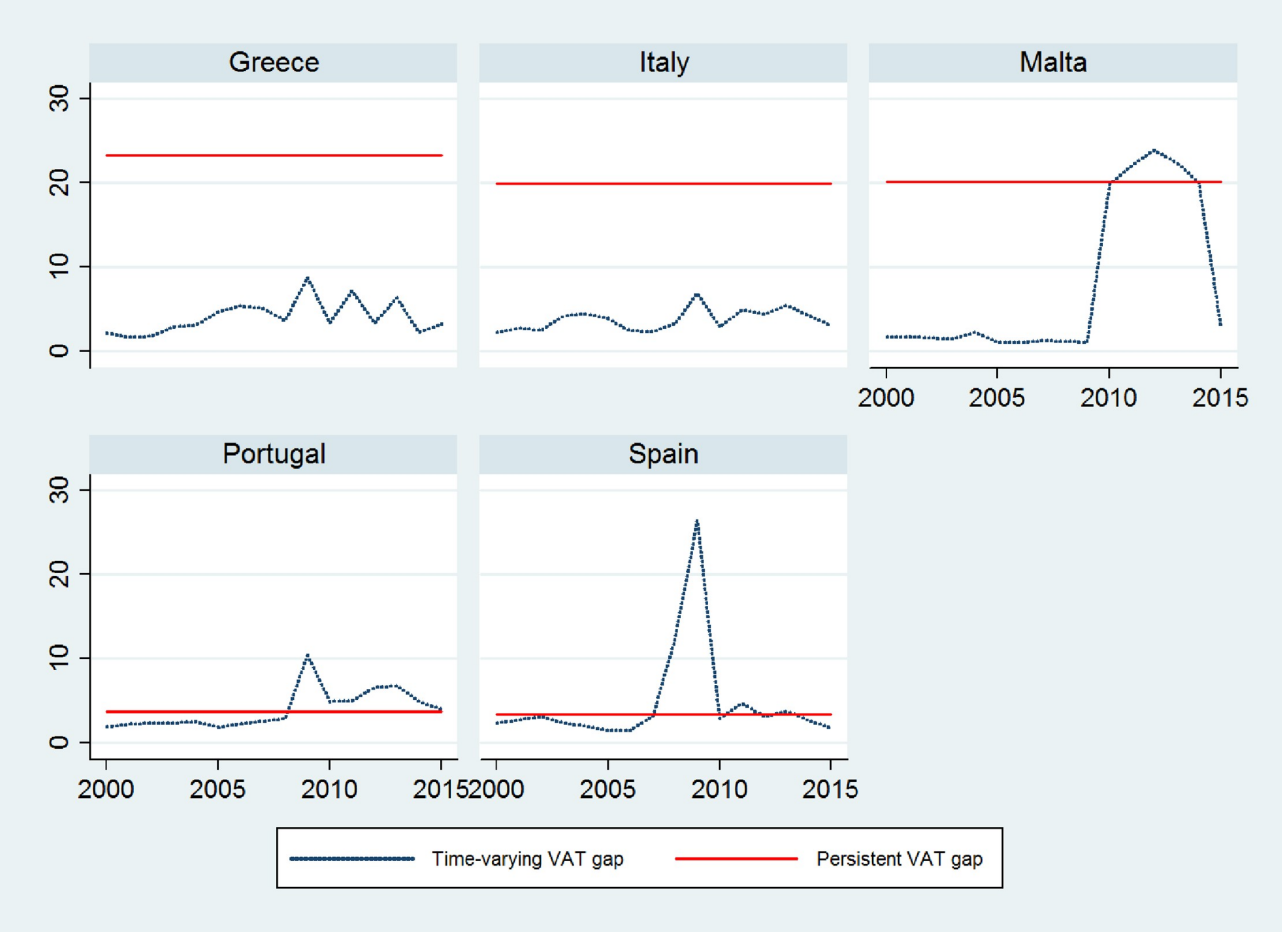
Time-varying VAT gap and persistent VAT gap in the southern group of EU countries. The blue dotted line represents the time-dependent Value Added Tax gap and the red line represents the country-specific or persistent Value Added Tax gap, between 2005–2015. The persistent and time-dependent VAT gap was generated in STATA after the STFM estimation.

Fig 2 presents the time-varying VAT gap and persistent VAT gap for the southern group of EU countries. In the southern group of EU countries, two states stand out by presenting a similar pattern as the one found in the western group of EU countries; these countries are Portugal and Spain, where time-varying and persistent VAT gap tend to overlap. This means that both tax agents’ experience and performance and tax morale contribute equally to the VAT gap.

In the case of the other three EU states, namely, Greece, Italy and Malta, there is a substantial difference between the persistent VAT gap and the time-varying VAT gap. This large difference shows that tax morale, coupled with complex tax laws (i.e., VAT tax regimes) contribute substantially to VAT gap.

**Fig 3:**
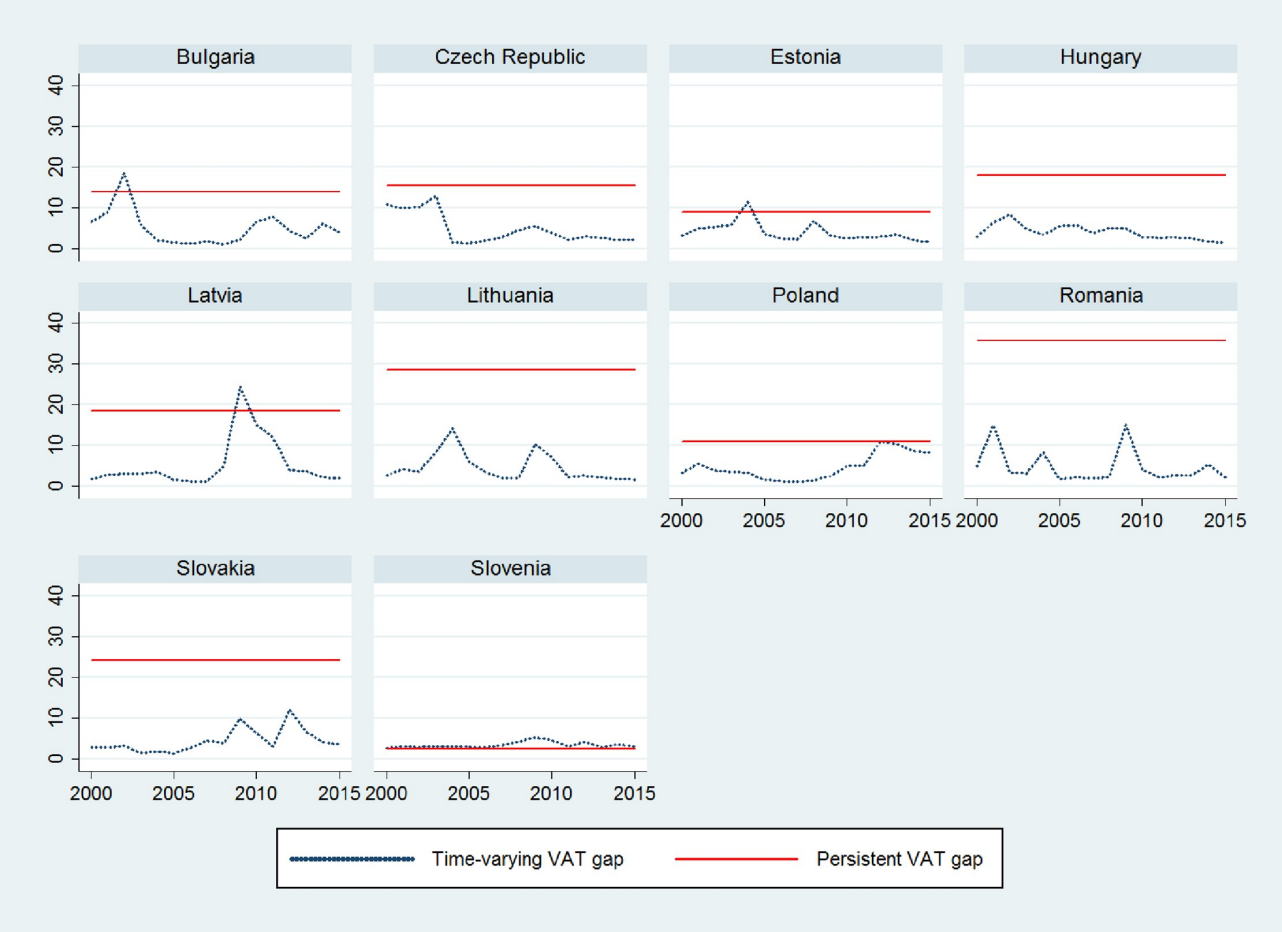
Time-varying VAT gap and persistent VAT gap in the eastern group of EU countries. The blue dotted line represents the time-dependent Value Added Tax gap and the red line represents the country-specific or persistent Value Added Tax gap, between 2005–2015. The persistent and time-dependent VAT gap was generated in STATA after the STFM estimation.

In the case of the eastern group of EU countries, as shows Fig 3, Slovenia is the only country that follows the pattern found in the corresponding western group, where the time-varying VAT gap equals the persistent VAT gap. Another pattern found is the moderate difference between time-varying VAT gap and persistent VAT gap in Bulgaria, the Czech Republic, Estonia and Poland. In these EU countries, the contribution of country-specific factors such as tax morale, tax law and tax administration organization tends to dominate the experience and performance of tax agents. On the other hand, we found large differences between the two VAT gaps, especially in Romania, Lithuania, Slovakia, Latvia and Hungary. The performance and experience of tax agents matters little compared to tax morale, the complexity of tax law, and the organization of tax administrations. Thus, to tackle VAT gap, especially the persistent VAT gap in the latter-discussed EU countries, there is a need to shape appropriate policy measures that would overcome the issues related to country-specific obstacles to VAT efficiency in particular and to tax revenue efficiency in general.

To compare the results of the top-down method adopted by CASE [4] and the STFM empirical analysis, we extract the results obtained from panel data analysis for 2015. As shown in Table 8, the VAT gap estimated by the STFM is notably different from the VAT gap estimates of CASE [4]. There are large differences between overall VAT gap and CASE VAT gap, especially in western EU countries, where the STFM-estimated VAT gap is on average 3–4% lower that VAT gap obtained from a top-down method. Comparing the results obtained from the STFM regarding the VAT gap in the UK in 2015, we obtained 6.81% while HMRC [14] estimated a VAT gap of 8.4%. The 1.59% difference can be attributed to methodological differences between HMRC [14] procedures to calculate the VAT gap and the STFM. As shown in Table 7, HMRC tends to overestimate the VAT gap due to the limitations of the top-down method. The overall image shows that the revised estimates by HMRC [14] are lower than the initial ones, which reinforces the assumption that the VAT gap estimates obtained from the STFM are more accurate than the top-down ones.

**Table 8:** Results from the panel data STFM estimation of VAT gap versus the VAT gap obtained by the CASE [4, 5] top-down method for 2015.

Country	CASE VAT gap 2015 in % of VTTL	CASE VAT gap 2015 in % of VTTL (revised)	Time-varying VAT gap in %	Persistent VAT gap in %	Overall VAT gap in %
Austria	**8.24**	**8**	2.70	3.79	**6.39**
Belgium	**10.76**	**10.77**	2.77	5.77	**8.38**
Bulgaria	**20.58**	**20.67**	4.04	13.95	**17.43**
Czech Republic	**16.48**	**16.92**	2.02	15.50	**17.21**
Denmark	**10.83**	**10.7**	3.59	3.66	**7.12**
Estonia	**4.88**	**6.33**	1.53	8.90	**10.29**
Finland	**6.95**	**6.89**	2.35	4.12	**6.37**
France	**11.71**	**11.58**	2.78	5.55	**8.17**
Germany	**9.56**	**10.45**	2.64	3.87	**6.40**
Greece	**28.27**	**29.37**	3.23	23.27	**25.75**
Hungary	**13.74**	**15.4**	1.35	18.03	**19.13**
Ireland	**9.94**	**10.61**	3.54	3.49	**6.91**
Italy	**25.78**	**26.13**	3.03	19.92	**22.34**
Latvia	**17.97**	**17.17**	1.97	18.44	**20.05**
Lithuania	**26.42**	**25.57**	1.64	28.46	**29.63**
Luxembourg	**5.56**	**2.28**	2.43	3.71	**6.05**
Malta	**22.54**	**3.42**	2.83	20.14	**22.40**
Netherlands	**7.94**	**9.49**	3.88	1.62	**5.44**
Poland	**24.51**	**24.3**	8.27	10.92	**18.29**
Portugal	**11.46**	**12.88**	3.99	3.71	**7.55**
Romania	**37.19**	**34.48**	2.18	35.69	**37.09**
Slovakia	**29.40**	**29.27**	3.57	24.19	**26.90**
Slovenia	**5.49**	**8.24**	2.97	2.39	**5.28**
Spain	**3.52**	**4.05**	1.78	3.43	**5.15**
Sweden	**-1.42**	**3.51**	2.10	1.07	**3.14**
United Kingdom	**10.88**	**11.04**	3.03	3.91	**6.81**
EU-26 average	**12.82**	**13.20**	2.93	11.06	**13.68**

Source: Own calculation and CASE [4, 5].

We found that countries such as Estonia, Hungary, Latvia, Lithuania, Luxembourg, Spain and Sweden have a higher VAT gap than the estimates obtained by CASE [4] for 2015. There are also similarities between the two methods regarding the obtained results. According to both the STFM and a top-down method, Sweden is the most efficient country in terms of VAT revenues, and Romania is the country with the largest VAT gap. Moreover, there are close matches regarding the overall VAT gap and CASE VAT gap in the case of the Czech Republic, Finland, Luxembourg, Malta, Romania and Slovenia, where the differences do not exceed 1%. Most notably, there is also a close match between the STFM and top-down method in terms of average VAT gap in the EU. Although the average overall VAT gap for EU-26 countries is higher under the STFM than the VAT gap under CASE [4], the difference is less than 1%. As noted in the second section of this paper, CASE [5] revised the VAT gap estimates for 2015. As shown in Table 8, according to CASE [5], Sweden is not the most efficient country in collecting VAT revenues; it is surpassed by Luxembourg. The revised VAT gap estimates for Sweden are very close to our VAT gap estimates using the STFM. Another surprising result obtained by CASE [5] was the revised estimated for Malta. While CASE [4] estimated a VAT gap of 22.54% in 2015, CASE [5] found that Malta had a VAT gap of only 3.42% for the same year. Overall, CASE [5] found that the VAT gap in the EU-28 is 13.2%, or half of a percent higher than the estimates of CASE [4]. CASE’s [5] revised overall VAT gap for the EU-28 is lower only by 0.48% than our average VAT gap for the EU-26 resulting from the STFM. However, the large differences for individual countries in VAT gap estimates between CASE [4] and CASE [5] raise questions regarding the accuracy of VAT gap estimates obtained from top-down method.

## Robustness check

This section investigates the robustness of our STF model. Karakaplan and Kutlu [56] underline that if the inputs used to estimate stochastic frontier models are correlated with the two-sided error term, this leads to biased estimates of technical efficiency. The same bias can arise from the correlation of the inefficiency term with the two-sided error term. Moreover, the issue of endogeneity appears to be more complex if one assumes the correlation between the environmental variables (*Z* variables) and the two-sided error term. Estimating a classical maximum likelihood model where endogenous variables are included leads to inconsistent estimates of technical efficiency. Karakaplan and Kutlu [56, 57] recommend a standard Instrumental Variables approach in order to test whether correction for endogeneity is needed.

Therefore, we adopt the methodology proposed by Karakaplan and Kutlu [56] to investigate whether endogeneity is present in our STF models. Karakaplan and Kutlu [56] estimate a stochastic model assuming that the set of explanatory variables includes both exogenous and endogenous variables. To test whether endogeneity is present, the authors assume that *v*_*i*_ is normally distributed with a mean adjusted by a correction term for bias *η*.

In the first endogeneity test, we investigate whether the VTTL is endogenous by running the SF model proposed by Karakaplan and Kutlu [56]. The instrumental variable chosen to test whether the VTTL is exogenous or endogenous is total population.

It is important to mention that the Karakaplan and Kutlu [56] model is a stochastic frontier estimation assuming a normal/half normal distribution. The first column of Table 9 presents the results for Model EX, which assumes that the included variables are exogenous. The second column presents the results for Model EN, where the explanatory variables are assumed to be endogenous. The null hypothesis of the endogeneity test states that the model does not need to be corrected for endogeneity. According to the eta (*η*) endogeneity test, we were unable to reject the null hypothesis, meaning that the VTTL is exogenous and no instrumental variables are needed to correct for endogeneity.

**Table 9:** Endogeneity test for the input variable of the stochastic tax frontier model.

Table: Estimation Results				
	Model EX		Model EN	
Dep.var: lnvat				
Constant	0.012	(-0.101)	-0.024	(-0.168)
lnvttl	0.994***	(-0.01)	0.998***	(-0.017)
Dep.var: ln(σ^2^_u)				
Constant	-3.355***	(-0.32)	-3.356***	(-0.316)
Dep.var: ln(σ^2^_v)				
Constant	-3.355***	(-0.32)		
Dep.var: ln(σ^2^_w)				
Constant			-5.391***	(-0.072)
eta1 (lnvttl)			(-0.005)	(-0.021)
**eta Endogeneity Test**			**X2 = 0.06**	**p = 0.808**
Observations	416		416	
Log Likelihood	484.89		27.04	
Mean Tech Efficiency	0.8713		0.8712	
Median Tech Efficiency	0.9193		0.916	

Source: Own calculation. Notes: Standard errors are in parentheses. Asterisks indicate significance at the 0.1% (***), 1% (**) and 5% (*) levels.

In the second test, we seek to investigate whether our environmental variables (*Z* variables) used to estimate the time-decay inefficiency model proposed by Battese and Coelli [7] are correlated with the two-sided error term. The instrument variable is total population, as in the previous test.

In the first column of Table 10, the exogenous stochastic model (Model EX) is estimated where the explanatory variables used in time-varying inefficiency model are assumed to be exogenous. The second column estimates the endogenous model (Model EN) where the *Z* variables are assumed to be correlated with the two-sided error term. The correction term for bias, *η*, is included in the latter model in order to adjust the mean of normally distributed *v*_*i*_. The eta (*η*) endogeneity test fails to reject the null hypothesis, meaning that the time-varying inefficiency model does not need correction for endogeneity.

**Table 10:** Endogeneity test for the environmental variables (*Z* variables) of the stochastic tax frontier model.

Table: Estimation Results				
	Model EX		Model EN	
Dep.var: lnvat				
Constant	0.343**	(-0.123)	0.415**	(-0.134)
lnvttl	0.963***	(-0.011)	0.956***	(-0.012)
Dep.var: ln(σ^2^_u)				
Constant	1.712	(-2.293)	4.756	(-3.18)
lncpi	-1.032***	(-0.268)	-1.417***	(-0.377)
lnshad	0.928*	(-0.393)	0.714	(-0.435)
lndocimp	0.732*	(-0.316)	0.777*	(-0.33)
lncostimp	-0.453	(-0.232)	-0.520*	(-0.22)
lntimeimp	-0.637	(-0.367)	-0.771*	(-0.363)
Dep.var: ln(σ^2^_v)				
Constant	-5.449***	(-0.073)		
Dep.var: ln(σ^2^_w)				
Constant			-5.461***	(-0.073)
eta1 (lncpi)			-0.074	(-0.055)
eta2 (lnshad)			-0.018	(-0.036)
eta3 (lndocimp)			0.008	(-0.022)
**eta Endogeneity Test**			**X2 = 1.83**	**p = 0.609**
Observations	416		416	
Log Likelihood	496.61		670.12	
Mean Tech Efficiency	0.8462		0.8378	
Median Tech Efficiency	0.8826		0.8734	

Source: Own calculation. Notes: Standard errors are in parentheses. Asterisks indicate significance at the 0.1% (***), 1% (**) and 5% (*) levels.

## Limitations of the study

In this section, we discuss the limitations of our study and the advantages of using the stochastic tax frontier model to estimate the VAT gap relative to a top-down method.

One of the limitations of this paper arises from the accuracy of the data used regarding the calculation of VAT total tax liability, because the CASE [1, 2, 3, 4] reports are based on data provided by Eurostat and the Own Resource Statements provided by each EU member state. The data used to calculate VTTL is prone to miscalculations due to the adjustments made in the process. Another limitation of this study is the reduced form of the STFM adopted, where only one input is used to estimate technical efficiency and VAT gap. The literature stresses that the STFM is shrouded in uncertainty compared to traditional stochastic production frontier models. In the case of the SFM, where company output efficiency is analyzed, there is no uncertainty regarding the accuracy of the included inputs (e.g., labor, capital and materials). However, there is an ongoing debate about the relevancy of inputs used in the case of the STFM.

Another limitation of this study is directly related to our objective: the estimation of VAT gap. Alfirman [38] emphasizes that the estimated tax efficiency levels and the resulting tax gaps between analyzed countries should be interpreted cautiously. Since the estimated technical inefficiency in traditional SFM means the failure of a company to produce the maximum output from a given set of inputs, this interpretation does not necessarily apply to STFM estimates. Therefore, the obtained levels of VAT technical efficiency and the resulting VAT gaps do not necessarily represent a “failure” of the EU member states to collect the maximum amount of VAT revenues. According to Alfirman [38], the lower level of VAT efficiency could also be attributed to the fact that some countries choose a lower level of taxation, which represents the country’s preference for a lower level of public goods and services and thus a smaller public sector. This preference is especially applicable to the Scandinavian countries, where the choice of a large public sector is reflected in high tax rates.

Furthermore, another notable limitation of this study stems from the limited number of external factors assumed to affect the inefficiency of VAT. Due to the complexities in Battese and Coelli’s [7] model, increasing the number of external factors restricts the model convergence.

With respect to comparative advantages, the STFM procedure to estimate technical efficiency and VAT gap in the EU countries offers an analytic advantage compared to a top-down method. First, the STFM provides more options for determining VAT gap and for investigating the main determinants of inefficiency related to VAT revenues. Second, the latest innovation regarding efficiency analysis through the SFM allows the user to disentangle country heterogeneity, time-varying inefficiency and country-specific or persistent inefficiency. The STFM is superior to a top-down method because the separation between time-varying and persistent inefficiency has different policy implications and could therefore be addressed from different perspectives. While time-varying inefficiency is caused by exogenous factors that are not necessarily country-specific and occur randomly, persistent inefficiency is country-specific and depends on particular issues that can be addressed by appropriate policy measures.

## Concluding remarks

In this paper, we pursued an alternative method to analyze the VAT gap. We adopted the stochastic tax frontier model, following the models proposed by Battese and Coelli [7] and Kumbhakar Lien and Hardaker [8]. The first model addresses the issue of heteroscedasticity, assuming that the VAT inefficiency is affected by external factors not included in either the inputs or outputs of the STFM. The second model addresses the issue of country heterogeneity and the separation between time-varying VAT inefficiency and persistent or country-specific VAT inefficiency. The estimated technical efficiency and VAT gap from both models prove to be slightly lower than the size of VAT gap found by CASE [1, 2, 3, 4]. The first advantage of the STFM over the top-down method adopted by CASE reports is the possibility of not only estimating the VAT gap but also analyzing what the external factors that affect the VAT inefficiency are. Therefore, this method represents a useful tool for policymakers, enabling them to identify appropriate measures to reduce tax avoidance and tax fraud in the area of VAT. Moreover, the large differences between CASE’s [4] VAT gap estimates and CASE’s [5] revised VAT gap estimates for 2015 raise concerns regarding the reliability of top-down estimates. The model of STFM proposed by Battese and Coelli [7] allowed us to find that CPI has a negative impact on VAT inefficiency, especially in the eastern group of EU countries. The shadow economy also tends to increase VAT inefficiency in the eastern EU. We found that the number of documents needed to import goods has a negative impact on VAT compliance and increases VAT inefficiency. We also found that as the cost of imported goods increases, VAT compliance will also increase. This leads us to conclusion that small value imports tend to go unreported, and as the value increases, the VAT inefficiency decreases. The second STFM model adopted found that the average time-varying VAT gap in the EU-26 countries is less than 5%, compared to country-specific or persistent VAT gap, which amounts to 11%. For 2015, we found that the average overall VAT gap in the EU-26 countries was 13.6%, which is only slightly higher than the CASE [4] estimates. Moreover, the average overall VAT gap for 2000–2015 was 14.73%, which is lower than the average estimates from the CASE reports.

The policy recommendations that can be extracted from this study are directly related to the results obtained by the two STF models adopted. The second STFM adopted in this paper, namely, the model proposed by Kumbhakar, Lien and Hardaker [8], allows us to estimate time-varying and persistent VAT gap in EU countries. A higher persistent VAT gap can be interpreted as consequence of complicated tax legislation, low tax enforcement, low tax morale, a large shadow economy and high bureaucracy. To identify appropriate policy recommendations, we use the output of our first STFM estimation, namely, the inefficiency model and the marginal effects estimates.

The western group of EU countries includes 11 member states that show a similar pattern in terms of VAT gap estimates. However, Belgium and France stand out from the western group of EU countries as their persistent VAT gap is considerably higher than their time-varying VAT gap. In this case, policymakers should aim to improve tax legislation and the organization of their tax revenue services before improving the efficiency of tax administrations in collecting the VAT revenues. In Austria, Denmark, Finland, Germany, Luxembourg and the UK, there are no significant differences between the time-dependent and country-specific VAT gap. Hence, these countries should aim to improve both their tax legislation and the organizational design of their revenue services as well as improving the efficiency of tax administrations. The Netherlands and Sweden show a different pattern of VAT gap evolution between 2000–2015. In these countries, the time-varying VAT gap is significantly higher than the persistent VAT gap. Therefore, the policymakers in these countries should focus more on the human factor inside their tax agencies in order to improve the efficiency of VAT revenues collection and reduce VAT gap.

In the southern group of EU countries, Portugal and Spain show a similar pattern of VAT gaps, where the differences between time-varying and persistent VAT gap are small. Thus, these countries should equally improve their tax legislation and the efficiency of tax administrations to tackle VAT noncompliance. The rest of the southern group of EU countries, namely, Greece, Italy and Malta, present a different pattern of VAT gap evolution. The persistent VAT gap is substantially higher than the time-varying VAT gap. At first glance, we observe that the inefficiency of tax administrations has little contribution to the large VAT gaps registered in Greece, Italy and Malta. According to our results, Greece needs to tackle corruption in order to decrease the VAT gap. Second, policymakers should also address the shadow economy, which contributes substantially to VAT gap occurrence. Tackling the shadow economy involves a complex set of measures that addresses tax morale and tax enforcement. A large shadow economy could represent a response to government intervention in economy, over-regulation and high tax burden. Greece should also address its large bureaucracy, which increases the cost of tax compliance and leads to uncollected VAT revenues. Malta should adopt a similar set of measures to Greece to increase tax compliance; the only difference is that the shadow economy has a higher marginal impact on VAT gap than corruption in Malta’s case. Hence, in order to decrease VAT gap in Malta, policymakers should tackle tax evasion and increase tax enforcement to decrease the undeclared economy. In case of Italy, the shadow economy and a large bureaucracy has a relatively low impact on VAT gap when compared with Greece and Malta. However, the main factor that enhances VAT gap occurrence, according to our marginal effects estimation, is the corruption perception level. Therefore, in order to increase tax compliance, policymakers in Italy should aim to decrease the corruption level.

Slovenia is the only country from the eastern group of EU member states that has a persistent and time-varying VAT gap similar to those of the western group of EU countries. There are almost no differences between time-dependent and country-specific VAT gaps, thus Slovenia should equally improve its tax legislation and tax administration efficiency to decrease the VAT gap. Bulgaria, the Czech Republic, Estonia, Hungary, Latvia and Poland show mid-level differences between time-varying and persistent VAT gap, with the latter being higher.

The worst performing EU countries in terms of VAT revenues collection, namely, Lithuania, Romania and Slovakia, have the largest differences between time-varying and persistent VAT gaps. In these countries, the inefficiency of their tax administrations matters little in comparison with the contribution of tax legislation, tax morale and tax enforcement to tax noncompliance. According to marginal effects estimation, Bulgaria, Estonia, Latvia, Lithuania and Romania should all aim to equally decrease the high level of corruption as well as implement measures intended to tackle the shadow economy in order to improve tax compliance. Consequently, structural measures are needed to improve tax enforcement and increase the quality of governance in order to enhance tax morale and decrease the shadow economy. Moreover, these EU states should tackle the negative impact of bureaucracy by tax law simplification in order to decrease the cost of tax compliance. In the case of the Czech Republic, Hungary, Poland and Slovakia, we found that corruption plays a central role in enhancing tax noncompliance and VAT gap occurrence. Only in Hungary and Poland are there some mild contributions of the shadow economy and a large bureaucracy to VAT gap occurrence. Hence, the main policy recommendation for these four EU member states is to implement corruption reduction measures in order to increase tax compliance. To a lesser extent, tax enforcement and tax law simplification should be worked toward along with improving tax morale in order to reduce tax noncompliance.

In conclusion, this paper shows that the STFM represents an appropriate alternative to existent methodologies used to estimate the VAT gap because this approach allows for the identification of the main enhancing factors of VAT inefficiency and the disentangling of time-varying and country-specific inefficiencies. A top-down method is useful and essential in theoretical tax liability calculation. However, this method should represent only the first step in a tax gap calculation. The second step should be given to the stochastic tax frontier model in order to produce accurate VAT gap estimates and analyze the main determinants of tax noncompliance. Based on this, policymakers may formulate more targeted measures to reduce the VAT gap. Comparing the estimates obtained by the STFM and the top-down method adopted by CASE [4, 5], we believe that the former approach improves the accuracy of the determination of VAT efficiency and VAT gap.

## Supporting information

S1 Appendix Contains **Table A.1** which shows the summary statistics of the variables used to estimate the STFM. Moreover, **Table A.2, A.3, A.4, A.5, A.6** and **A.7** show the confidence intervals regarding the VAT gap estimates and technical efficiency of VAT.
(DOCX)

S2 Appendix Contains **Graph A, Graph B** and **Graph C** representing the marginal effects of CPI, shadow economy, documents needed to import and cost to import on the mean inefficiency E(u) in the eastern group of EU countries, in the southern Group of EU countries and the western group of EU countries.
(DOCX)

S1 Dataset Contains the data used in our STFM. Namely, we include data regarding the VAT, the VTTL, the *Z* variables and the Instrumental Variable–total population. The data can be found in the first Excel sheet, entitled “Raw data used for STFM”. Furthermore, we include two more sheets in the same Excel document where the latest VAT gap estimates are presented, obtained from CASE [5] and HMRC [14].
(XLSX)
