# Supplementary material for: Alternative method to measure the VAT gap in the EU: Stochastic tax frontier model approach
Source: PLoS One. 2019 Jan 28;14(1):e0211317. doi: 10.1371/journal.pone.0211317 (PMC6349340; doi:10.1371/journal.pone.0211317)
Supplement: S1 Appendix — Contains Table A.1 which shows the summary statistics of the variables used to estimate the STFM. Moreover, Table A.2, A.3, A.4, A.5, A.6 and A.7 show the confidence intervals regarding the VAT gap estimates and technical efficiency of VAT. (DOCX) [file pone.0211317.s001.docx]

**S1 Appendix**

Table A.1. The summary statistics of the variables used to estimate the STFM

| Variable | Obs. | Mean | Std. Dev. | Min | Max |
| --- | --- | --- | --- | --- | --- |
| VAT | 416 | 31524.77 | 45324.26 | 236 | 211616 |
| VTTL | 416 | 36857.85 | 52426.47 | 283 | 233982 |
| CPI | 416 | 63.9976 | 18.61513 | 26 | 100 |
| Shadow Economy | 416 | 20.07837 | 7.333486 | 7.5 | 36.9 |
| Documents needed to import | 416 | 4.803953 | 1.795004 | 2 | 15 |
| Time to import | 416 | 11.95246 | 5.246975 | 5 | 28 |
| Cost to import | 416 | 1187.19 | 324.522 | 489 | 2401 |
| Population | 416 | 1.90e+07 | 2.30E+07 | 390077.1 | 82500000 |

Source: CASE [1, 2, 3, 4], Schneider [45, 46], Transparency International Report, Doing Business of World Bank and World Development Indicators. The VAT and VTTL are expressed in Million EUR. Shadow economy is expressed as percent of GDP; Documents needed to import goods and services are expressed in number of documents; Time to import is expressed in days and cost to import is expressed in US Dollars for each container; Population is expressed in millions.

Table A.2. Average overall VAT gap with mean, lower bound and upper bound at 95% level

| Country | Average Overall VAT gap (Mean) | Average Overall VAT gap (Lower Bound 95%) | Average Overall VAT gap (Lower Bound 95%) |
| --- | --- | --- | --- |
| Austria | 7.11 | 0.49 | 16.68 |
| Belgium | 9.04 | 1.48 | 18.64 |
| Bulgaria | 18.30 | 10.59 | 26.84 |
| Czech Republic | 19.55 | 12.05 | 27.90 |
| Denmark | 6.91 | 0.44 | 16.48 |
| Estonia | 12.54 | 4.74 | 21.73 |
| Finland | 7.67 | 0.62 | 17.24 |
| France | 8.95 | 1.36 | 18.52 |
| Germany | 7.16 | 0.50 | 16.75 |
| Greece | 26.39 | 19.68 | 34.23 |
| Hungary | 21.32 | 14.17 | 29.71 |
| Ireland | 7.21 | 0.51 | 16.65 |
| Italy | 22.92 | 16.10 | 31.14 |
| Latvia | 22.86 | 16.00 | 30.68 |
| Lithuania | 31.80 | 25.48 | 38.95 |
| Luxembourg | 7.40 | 0.55 | 16.89 |
| Malta | 26.41 | 19.98 | 33.33 |
| Netherlands | 5.09 | 0.21 | 13.73 |
| Poland | 15.14 | 7.07 | 24.09 |
| Portugal | 7.54 | 0.66 | 16.95 |
| Romania | 38.79 | 33.22 | 45.18 |
| Slovakia | 27.53 | 20.86 | 35.20 |
| Slovenia | 5.74 | 0.26 | 14.91 |
| Spain | 8.05 | 1.84 | 16.95 |
| Sweden | 4.44 | 0.17 | 12.43 |
| United Kingdom | 7.20 | 0.51 | 16.79 |
| Average EU | 14.73 | 8.06 | 23.41 |

Source: Own calculation.

Table A.3. Average persistent VAT gap with mean, lower bound and upper bound at 95% level

| Country | Average Persistent VAT gap (Mean) | Average Persistent VAT gap (Lower Bound 95%) | Average Persistent VAT gap (Upper Bound 95%) |
| --- | --- | --- | --- |
| Austria | 3.79 | 0.34 | 8.06 |
| Belgium | 5.77 | 1.34 | 10.18 |
| Bulgaria | 13.95 | 9.68 | 18.02 |
| Czech Republic | 15.50 | 11.30 | 19.50 |
| Denmark | 3.66 | 0.31 | 7.90 |
| Estonia | 8.90 | 4.37 | 13.21 |
| Finland | 4.12 | 0.44 | 8.44 |
| France | 5.55 | 1.18 | 9.96 |
| Germany | 3.87 | 0.37 | 8.15 |
| Greece | 23.27 | 19.45 | 26.90 |
| Hungary | 18.03 | 13.95 | 21.91 |
| Ireland | 3.49 | 0.27 | 7.69 |
| Italy | 19.92 | 15.94 | 23.71 |
| Latvia | 18.44 | 14.39 | 22.30 |
| Lithuania | 28.46 | 24.91 | 31.85 |
| Luxembourg | 3.71 | 0.32 | 7.96 |
| Malta | 20.14 | 16.17 | 23.92 |
| Netherlands | 1.62 | 0.06 | 4.74 |
| Poland | 10.92 | 6.50 | 15.14 |
| Portugal | 3.71 | 0.32 | 7.96 |
| Romania | 35.69 | 32.49 | 38.73 |
| Slovakia | 24.19 | 20.42 | 27.78 |
| Slovenia | 2.39 | 0.11 | 6.14 |
| Spain | 3.43 | 0.26 | 7.61 |
| Sweden | 1.07 | 0.03 | 3.45 |
| United Kingdom | 3.91 | 0.38 | 8.19 |
| Average EU | 11.06 | 7.51 | 14.98 |

Source: Own calculation.

Table A.4. Average time dependent VAT gap with mean, lower bound and upper bound at 95% level

| Country | Average Time dependent VAT gap (Mean) | Average Time dependent VAT gap (Lower Bound 95%) | Average Time dependent VAT gap (Upper Bound 95%) |
| --- | --- | --- | --- |
| Austria | 3.44 | 0.14 | 9.37 |
| Belgium | 3.47 | 0.14 | 9.41 |
| Bulgaria | 5.05 | 1.02 | 10.75 |
| Czech Republic | 4.79 | 0.85 | 10.44 |
| Denmark | 3.38 | 0.13 | 9.31 |
| Estonia | 3.99 | 0.38 | 9.82 |
| Finland | 3.71 | 0.18 | 9.62 |
| France | 3.60 | 0.19 | 9.51 |
| Germany | 3.43 | 0.14 | 9.36 |
| Greece | 4.06 | 0.28 | 10.03 |
| Hungary | 4.02 | 0.26 | 9.99 |
| Ireland | 3.85 | 0.24 | 9.71 |
| Italy | 3.75 | 0.19 | 9.75 |
| Latvia | 5.41 | 1.89 | 10.78 |
| Lithuania | 4.66 | 0.76 | 10.41 |
| Luxembourg | 3.84 | 0.23 | 9.71 |
| Malta | 7.85 | 4.54 | 12.37 |
| Netherlands | 3.53 | 0.15 | 9.44 |
| Poland | 4.73 | 0.61 | 10.55 |
| Portugal | 3.98 | 0.34 | 9.77 |
| Romania | 4.82 | 1.07 | 10.53 |
| Slovakia | 4.41 | 0.55 | 10.27 |
| Slovenia | 3.44 | 0.14 | 9.34 |
| Spain | 4.78 | 1.59 | 10.11 |
| Sweden | 3.41 | 0.14 | 9.30 |
| United Kingdom | 3.43 | 0.14 | 9.36 |
| Average EU | 4.19 | 0.63 | 9.96 |

Source: Own calculation.

Table A.5. Average time dependent technical efficiency of VAT with mean, lower bound and upper bound at 95% level

| Country | Average Time dependent technical efficiency of VAT (Mean) | Average Time dependent technical efficiency of VAT (Lower Bound 95%) | Average Time dependent technical efficiency of VAT (Upper Bound 95%) |
| --- | --- | --- | --- |
| Austria | 96.56 | 90.63 | 99.86 |
| Belgium | 96.53 | 90.59 | 99.86 |
| Bulgaria | 94.95 | 89.25 | 98.98 |
| Czech Republic | 95.21 | 89.56 | 99.15 |
| Denmark | 96.62 | 90.69 | 99.87 |
| Estonia | 96.01 | 90.18 | 99.62 |
| Finland | 96.29 | 90.38 | 99.82 |
| France | 96.40 | 90.49 | 99.81 |
| Germany | 96.57 | 90.64 | 99.86 |
| Greece | 95.94 | 89.97 | 99.72 |
| Hungary | 95.98 | 90.01 | 99.74 |
| Ireland | 96.15 | 90.29 | 99.76 |
| Italy | 96.25 | 90.25 | 99.81 |
| Latvia | 94.59 | 89.22 | 98.11 |
| Lithuania | 95.34 | 89.59 | 99.24 |
| Luxembourg | 96.16 | 90.29 | 99.77 |
| Malta | 92.15 | 87.63 | 95.46 |
| Netherlands | 96.47 | 90.56 | 99.85 |
| Poland | 95.27 | 89.45 | 99.39 |
| Portugal | 96.02 | 90.23 | 99.66 |
| Romania | 95.18 | 89.47 | 98.93 |
| Slovakia | 95.59 | 89.73 | 99.45 |
| Slovenia | 96.56 | 90.66 | 99.86 |
| Spain | 95.22 | 89.89 | 98.41 |
| Sweden | 96.59 | 90.70 | 99.86 |
| United Kingdom | 96.57 | 90.64 | 99.86 |
| Average EU | 95.81 | 90.04 | 99.37 |

Source: Own calculation.

Table A.6. Average time dependent technical efficiency of VAT with mean, lower bound and upper bound at 95% level

| Country | Average Persistent technical efficiency of VAT (Mean) | Average Persistent technical efficiency of VAT (Lower Bound 95%) | Average Persistent technical efficiency of VAT (Upper Bound 95%) |
| --- | --- | --- | --- |
| Austria | 96.21 | 91.94 | 99.66 |
| Belgium | 94.23 | 89.82 | 98.66 |
| Bulgaria | 86.05 | 81.98 | 90.32 |
| Czech Republic | 84.50 | 80.50 | 88.70 |
| Denmark | 96.34 | 92.10 | 99.69 |
| Estonia | 91.10 | 86.79 | 95.63 |
| Finland | 95.88 | 91.56 | 99.56 |
| France | 94.45 | 90.04 | 98.82 |
| Germany | 96.13 | 91.85 | 99.63 |
| Greece | 76.73 | 73.10 | 80.55 |
| Hungary | 81.97 | 78.09 | 86.05 |
| Ireland | 96.51 | 92.31 | 99.73 |
| Italy | 80.08 | 76.29 | 84.06 |
| Latvia | 81.56 | 77.70 | 85.61 |
| Lithuania | 71.54 | 68.15 | 75.09 |
| Luxembourg | 96.29 | 92.04 | 99.68 |
| Malta | 79.86 | 76.08 | 83.83 |
| Netherlands | 98.38 | 95.26 | 99.94 |
| Poland | 89.08 | 84.86 | 93.50 |
| Portugal | 96.29 | 92.04 | 99.68 |
| Romania | 64.31 | 61.27 | 67.51 |
| Slovakia | 75.81 | 72.22 | 79.58 |
| Slovenia | 97.61 | 93.86 | 99.89 |
| Spain | 96.57 | 92.39 | 99.74 |
| Sweden | 98.93 | 96.55 | 99.97 |
| United Kingdom | 96.09 | 91.81 | 99.62 |
| Average EU | 88.94 | 85.02 | 92.49 |

Source: Own calculation.

Table A.7. Average overall technical efficiency of VAT with mean, lower bound and upper bound at 95% level

| Country | Average overall technical efficiency of VAT (Mean) | Average overall technical efficiency of VAT (Lower Bound 95%) | Average overall technical efficiency of VAT (Upper Bound 95%) |
| --- | --- | --- | --- |
| Austria | 92.89 | 83.32 | 99.51 |
| Belgium | 90.96 | 81.36 | 98.52 |
| Bulgaria | 81.70 | 73.16 | 89.41 |
| Czech Republic | 80.45 | 72.10 | 87.95 |
| Denmark | 93.09 | 83.52 | 99.56 |
| Estonia | 87.46 | 78.27 | 95.26 |
| Finland | 92.33 | 82.76 | 99.38 |
| France | 91.05 | 81.48 | 98.64 |
| Germany | 92.84 | 83.25 | 99.50 |
| Greece | 73.61 | 65.77 | 80.32 |
| Hungary | 78.68 | 70.29 | 85.83 |
| Ireland | 92.79 | 83.35 | 99.49 |
| Italy | 77.08 | 68.86 | 83.90 |
| Latvia | 77.14 | 69.32 | 84.00 |
| Lithuania | 68.20 | 61.05 | 74.52 |
| Luxembourg | 92.60 | 83.11 | 99.45 |
| Malta | 73.59 | 66.67 | 80.02 |
| Netherlands | 94.91 | 86.27 | 99.79 |
| Poland | 84.86 | 75.91 | 92.93 |
| Portugal | 92.46 | 83.05 | 99.34 |
| Romania | 61.21 | 54.82 | 66.78 |
| Slovakia | 72.47 | 64.80 | 79.14 |
| Slovenia | 94.26 | 85.09 | 99.74 |
| Spain | 91.95 | 83.05 | 98.16 |
| Sweden | 95.56 | 87.57 | 99.83 |
| United Kingdom | 92.80 | 83.21 | 99.49 |
| Average EU | 85.27 | 76.59 | 91.94 |

Source: Own calculation.
