# Supplementary material for: Alternative method to measure the VAT gap in the EU: Stochastic tax frontier model approach
Source: PLoS One. 2019 Jan 28;14(1):e0211317. doi: 10.1371/journal.pone.0211317 (PMC6349340; doi:10.1371/journal.pone.0211317)
Supplement: S2 Appendix — Contains Graph A, Graph B and Graph C representing the marginal effects of CPI, shadow economy, documents needed to import and cost to import on the mean inefficiency E(u) in the eastern group of EU countries, in the southern Group of EU countries and the western group of EU countries. (DOCX) [file pone.0211317.s002.docx]

**S2 Appendix**

**Graph A.** The marginal effects of CPI, shadow economy, documents required to import and cost of import on the mean inefficiency *E(u)* in the eastern group of EU countries


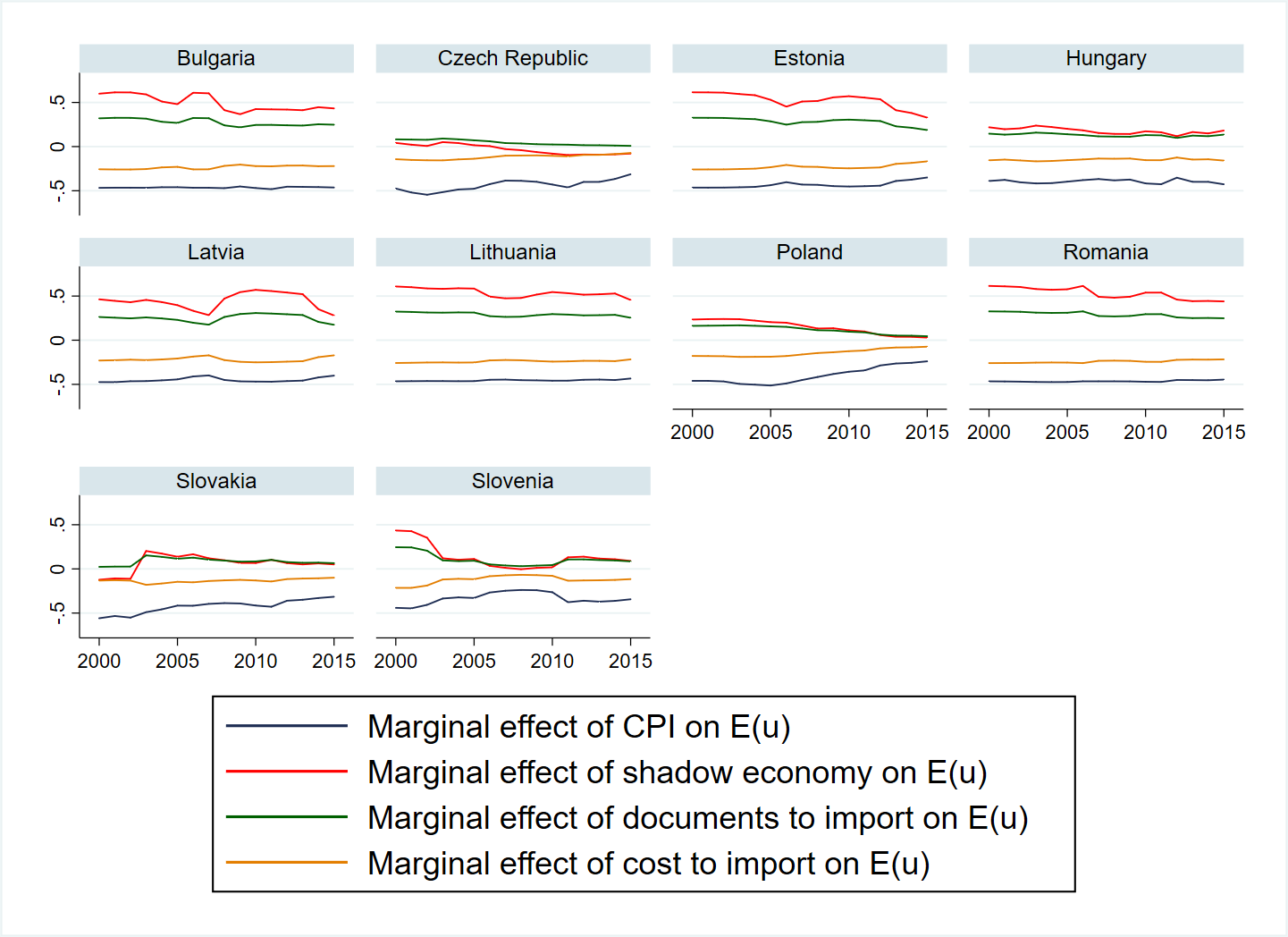


Source: Own compilation.

**Graph B.** The marginal effects of CPI, shadow economy, documents required to import and cost of import on the mean inefficiency *E(u)* in the southern group of EU countries


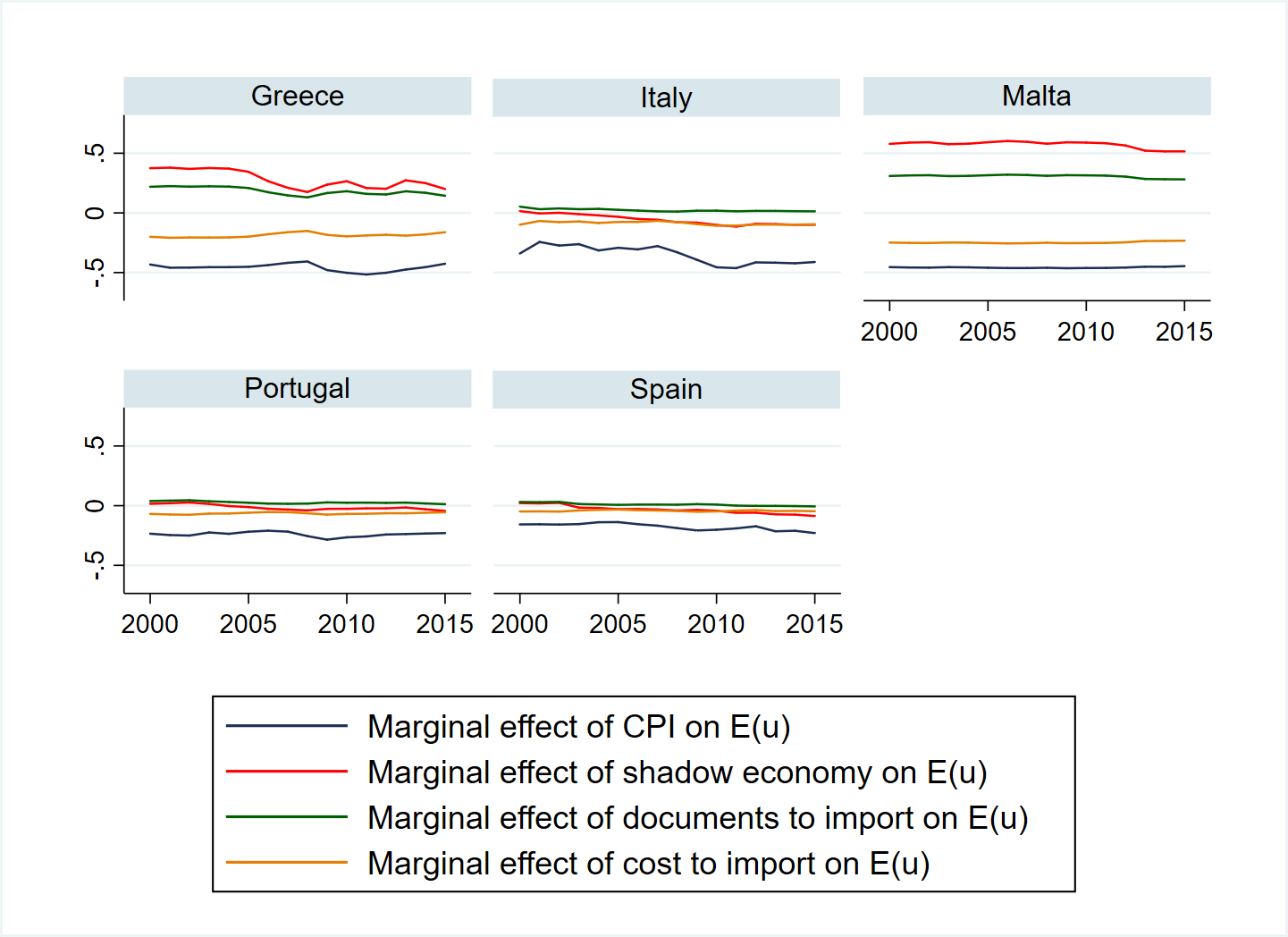


Source: Own compilation.

**Graph C.** The marginal effects of CPI, shadow economy, documents required to import and cost of import on the mean inefficiency *E(u)* in the western group of EU countries


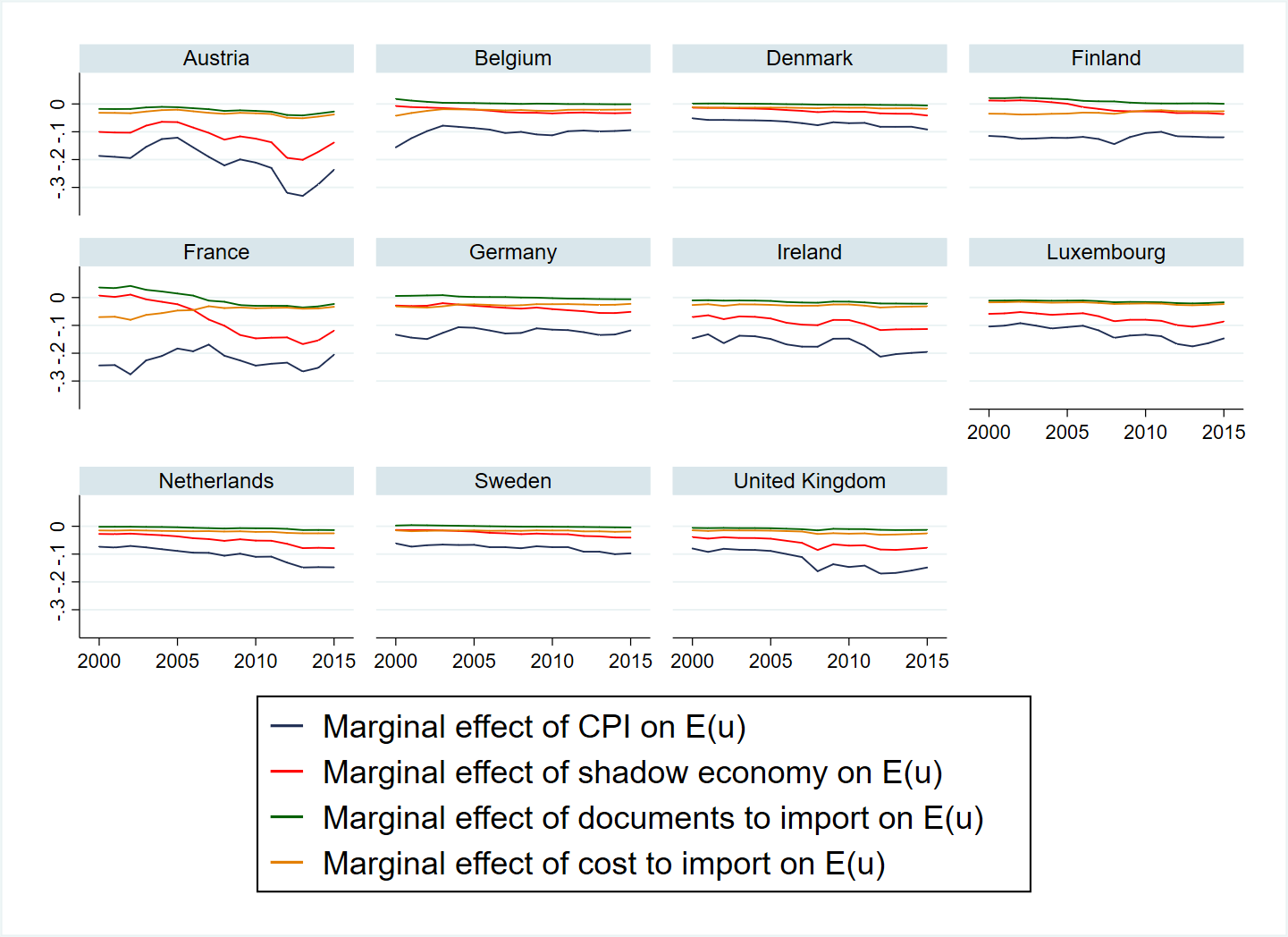


Source: Own compilation.
